# Bioenergetic Materials for Tissue Regeneration: Modulating Metabolism to Promote Cellular Anabolism

**DOI:** 10.1002/advs.77905

**Published:** 2026-09-27

**Authors:** Yuchen He, Yuwen Wang, Jonathan F. Gong, Yiting Lei, Fei Jin, Weihong Zhu, Rocky S. Tuan, Zhong Alan Li

**Affiliations:** ^1^ Department of Orthopaedics The Second Xiangya Hospital of Central South University Changsha Hunan China; ^2^ National Clinical Research Center for Metabolic Diseases Key Laboratory of Diabetes Immunology (Central South University) Ministry of Education, and Department of Metabolism and Endocrinology The Second Xiangya Hospital of Central South University Changsha Hunan China; ^3^ Department of Biomedical Engineering The Chinese University of Hong Kong Hong Kong SAR China; ^4^ School of Medicine University of California San Diego California USA; ^5^ Department of Orthopedic Surgery The First Affiliated Hospital of Chongqing Medical University Chongqing China; ^6^ Institute for Tissue Engineering and Regenerative Medicine School of Biomedical Sciences The Chinese University of Hong Kong Hong Kong SAR China; ^7^ Shun Hing Institute of Advanced Engineering (SHIAE) The Chinese University of Hong Kong Hong Kong SAR China; ^8^ Key Laboratory of Regenerative Medicine School of Biomedical Sciences Ministry of Education The Chinese University of Hong Kong Hong Kong SAR China; ^9^ Shenzhen Research Institute The Chinese University of Hong Kong Shenzhen China

**Keywords:** bioactive scaffold, bioenergetic materials, cellular bioenergetics, metabolism, tissue engineering, tissue regeneration

## Abstract

Tissue regeneration is an energy‐demanding process that requires adequate ATP production to support cellular proliferation, biosynthesis, and tissue remodeling. Under pathological and age‐related conditions, bioenergetic deficits impair intrinsic regenerative capacity by disrupting mitochondrial function, redox homeostasis, and anabolic signaling. Although conventional scaffold‐ and cell‐based bioengineering strategies have advanced regenerative medicine, they generally do not directly address the metabolic dysfunction that limits tissue repair. Recently, bioenergetic materials (BEMs) have emerged as a novel class of biomaterials engineered to modulate cellular metabolism in situ. By supplying tricarboxylic acid (TCA) cycle intermediates, delivering metabolic enzymes, or incorporating oxygen‐releasing and energy‐harvesting components, BEMs replenish cellular ATP, restore redox balance, and activate anabolic signaling pathways that support tissue regeneration. In this review, we classify BEMs according to their principal mechanisms of action and summarize recent advances in their application to bone, cartilage, skin, and neural tissue repair. We further discuss the major challenges to clinical translation and highlight future opportunities for integrating metabolic modulation with advanced fabrication techniques and smart, feedback‐regulated biomaterial systems. By reshaping the local metabolic microenvironments, BEMs represent a promising strategy to enhance tissue regeneration in pathological settings characterized by mitochondrial dysfunction, redox imbalance, inflammation, and insufficient energy supply.

## Graphical abstract

Bioenergetic materials (BEMs) modulate cellular metabolism to promote tissue regeneration. Their principal mechanisms of action include metabolic substrate supplementation, metabolic enzyme modulation, organelle transfer, electrical current regulation, redox homeostasis modulation, and oxygen tension modulation. By replenishing ATP, restoring redox homeostasis, and activating anabolic signaling, BEMs support the regeneration of diverse tissue types.

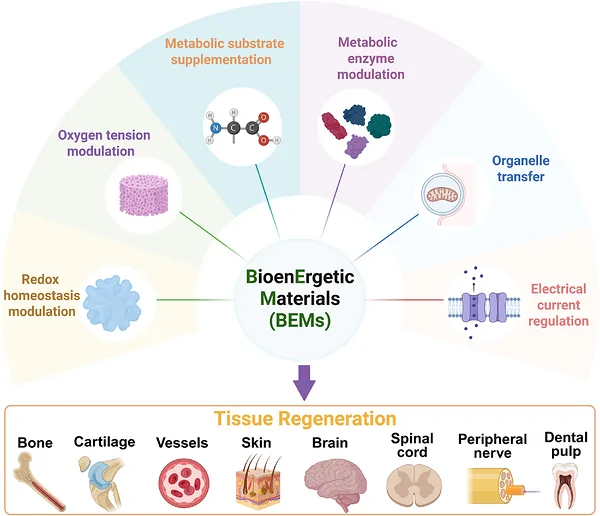

**Abbreviations**
3D: Three‐dimensional
ACKR1: Atypical chemokine receptor 1
Akt: Protein kinase B
ALDH2: Aldehyde dehydrogenase 2
AMPK: AMP‐activated protein kinase
Ang II: Angiotensin II
Ang‐1/2: Angiopoietin 1/2
AONFH: Alcohol‐associated ONFH
ASCs: Adipose stem cells
BAH: Bioenergetic‐active hydrogel
Bax: Bcl‐2‐associated X protein
BBB: Blood‐brain barrier
BCAA: Branched‐chain amino acid
Bcl‐2: B‐cell lymphoma 2
BEMs: Bioenergetic materials
bFGF: basic fibroblast growth factor
BGS: Bioactive glass scaffold
BMP2: Bone morphogenetic protein 2
BMSC: Bone marrow‐derived mesenchymal stem cell
CBE: Cellular bioenergetics
CCN2: Cellular communication network factor 2
CI: Confidence interval
CMC/ALG: Carboxymethyl chitosan/alginate
CNS: central nervous system
DKK1: Dickkopf‐1
DMM: Destabilization of the medial meniscus
ECM: extracellular matrix
ECs: Endothelial cells
EPCs: Endothelial progenitor cells
ER: Endoplasmic reticulum
ESWT: Extracorporeal shockwave treatment
EVs: Extracellular vesicles
FLSs: Fibroblast‐like synoviocytes
GABA: γ‐aminobutyric acid
GAPDH: glyceraldehyde‐3‐phosphate dehydrogenase
GONFH: Glucocorticoid‐induced ONFH
GOx: Glucose oxidase
HBOT: Hyperbaric oxygen therapy
HIFH: High‐energy intermediate fructose hydrogel
HUVECs: Human umbilical vein endothelial cells
IFN: Interferons
IP3R: Inositol 1,4,5‐trisphosphate receptor
iPSCs: Induced pluripotent stem cell
LCPD: Legg‐Calvé‐Perthes disease
LIPUS: Low‐intensity pulsed ultrasound
LNPs: Lipid nanoparticles
MDVs: Mitochondria‐derived vesicles
MEF2C: Myocyte enhancer factor 2C
MOF: Metal‐organic framework
MSCs: Mesenchymal stem cells
mTOR: Mammalian target of rapamycin
NAMPT: Nicotinamide phosphoribosyl transferase
NIR: Near‐infrared irradiation
NTUs: nanothylakoid units
OA: Osteoarthritis
OARSI: Osteoarthritis Research Society International
ONFH: Osteonecrosis of femoral head
OoCs: organs‐on‐chips
OXPHOS: Oxidative phosphorylation
PDGF‐BB: Platelet‐derived growth factor BB
PF‐4: Platelet factor‐4
PI3K: Phosphoinositide 3‐kinase
PLA: Polylactide
PLLA: Poly‐l‐lactic acid
PRP: Platelet‐rich plasma
PSer: Phosphoserine
RA: Rheumatoid arthritis
RyR2: Ryanodine receptor 2
SMSCs: Synovial mesenchymal stem cells
STAT1: Signal transducer and activator of transcription 1
TAZ: Transcriptional coactivator with PDZ‐binding motif
TCA: Tricarboxylic acid
TGF‐β: Transforming growth factor beta
TiOx: Titanium oxide
TNTs: Tunneling nanotubes
TSP‐1: Thrombospondin‐1
VCAM‐1: Vascular cell adhesion molecule 1
VEGF: Vascular endothelial growth factor.
α‐KG: α‐ketoglutarate

## 1 Introduction

Cellular metabolism is governed by a tightly coordinated balance between catabolism, which degrades macromolecules to generate energy, and anabolism, the energy‐dependent synthesis of cellular constituents [1]. Beyond sustaining cellular bioenergetics, metabolism is now recognized as a fundamental regulator of diverse signaling networks that coordinate both biochemical and non‐metabolic processes [2]. Metabolic intermediates function as more than just substrates for biosynthetic processes. They also serve as signaling molecules that regulate nutrient sensing, energy storage, cell survival, lineage specification, immune responses, extracellular matrix (ECM) synthesis, cytokine secretion, and tumor initiation and progression [3, 4, 5, 6, 7]. Accordingly, the dynamic equilibrium between catabolic and anabolic metabolism is essential for maintaining tissue homeostasis, whereas disruption of this balance contributes to aging, frailty, and impaired regenerative capacity [8, 9].

Although aging and cellular senescence are phenotypically heterogeneous processes, metabolic dysregulation remains a recognized hallmark of both [10, 11, 12]. Age‐associated alterations in nutrient‐sensing and metabolic pathways contribute to tissue degeneration and the development of chronic diseases [13, 14]. Importantly, these broad metabolic disturbances should be distinguished from bioenergetic dysfunction, which specifically refers to impaired cellular energy transduction, including mitochondrial respiratory dysfunction, reduced ATP production, disrupted tricarboxylic acid (TCA)‐coupled electron transport, oxygen/redox imbalance, abnormal mitochondrial dynamics, and defective membrane potential‐ or bioelectrical cue‐dependent repair responses [15, 16]. Accordingly, bioenergetic modulation represents a distinct therapeutic strategy that directly targets the processes governing cellular energy production, transfer, and utilization to support cell survival, proliferation, extracellular matrix synthesis, and tissue repair. Restoration of cellular bioenergetics has therefore emerged as a promising regenerative strategy, supported by encouraging preclinical and early clinical evidence [17, 18]. Nevertheless, most conventional regenerative approaches, including growth factor delivery and stem cell transplantation, primarily provide exogenous biological cues or structural support, without directly correcting the intrinsic bioenergetic deficits that limit endogenous repair [19]. Consequently, restoring cellular bioenergetic competence is increasingly important for unlocking the intrinsic regenerative capacity of host tissues.

To address these intrinsic bioenergetic deficits, bioenergetic materials (BEMs) have emerged as a distinct class of biomaterials designed to regulate the production, transfer, and utilization of biological energy, as well as the local metabolic conditions that govern these processes during tissue repair [20]. A seminal example was reported by Liu and coworkers, who introduced the concept of a bioenergetic‐active material scaffold [20]. This BEM scaffold is capable of releasing metabolically active TCA cycle intermediates, which are internalized by resident cells to augment mitochondrial membrane potential, enhance ATP generation, and accelerate bone regeneration in a critical‐sized defect model. In this review, we use the term BEM as an operational umbrella concept encompassing biomaterial systems that directly or indirectly regulate cellular bioenergetics to promote tissue regeneration, with bioenergetic activity supported by measurable changes in cellular energy status or energy‐generating processes, such as ATP production, mitochondrial membrane potential, mitochondrial respiration/oxidative phosphorylation, or energy‐related metabolic flux. Importantly, materials that alter ROS levels or antioxidant activity, glucose metabolism, metabolic gene expression, mitochondrial morphology or dynamics, or inflammatory responses are considered outside the scope of BEMs unless these effects are accompanied by experimentally demonstrated changes in cellular bioenergetics. Accordingly, redox regulation is included within BEMs only when it is functionally linked to energy production or mitochondrial bioenergetic activity. For example, piezoelectric hydrogels that convert physiological mechanical stimuli into localized bioelectrical signals to restore ATP production and promote the osteogenic differentiation of inflammatory periodontal ligament stem cells (PDLSCs) are included within the BEM scope [21]. Unlike conventional regenerative strategies that primarily rely on predefined biochemical or structural cues, BEMs can dynamically couple endogenous biological activities with bioenergetic regulation, enabling adaptive and feedback‐responsive interactions with the evolving tissue microenvironment [20, 21]. These self‐regulating properties position BEMs as a promising biomaterial platform for overcoming bioenergetic barriers and restoring endogenous regenerative capacity.

**FIGURE 1:**
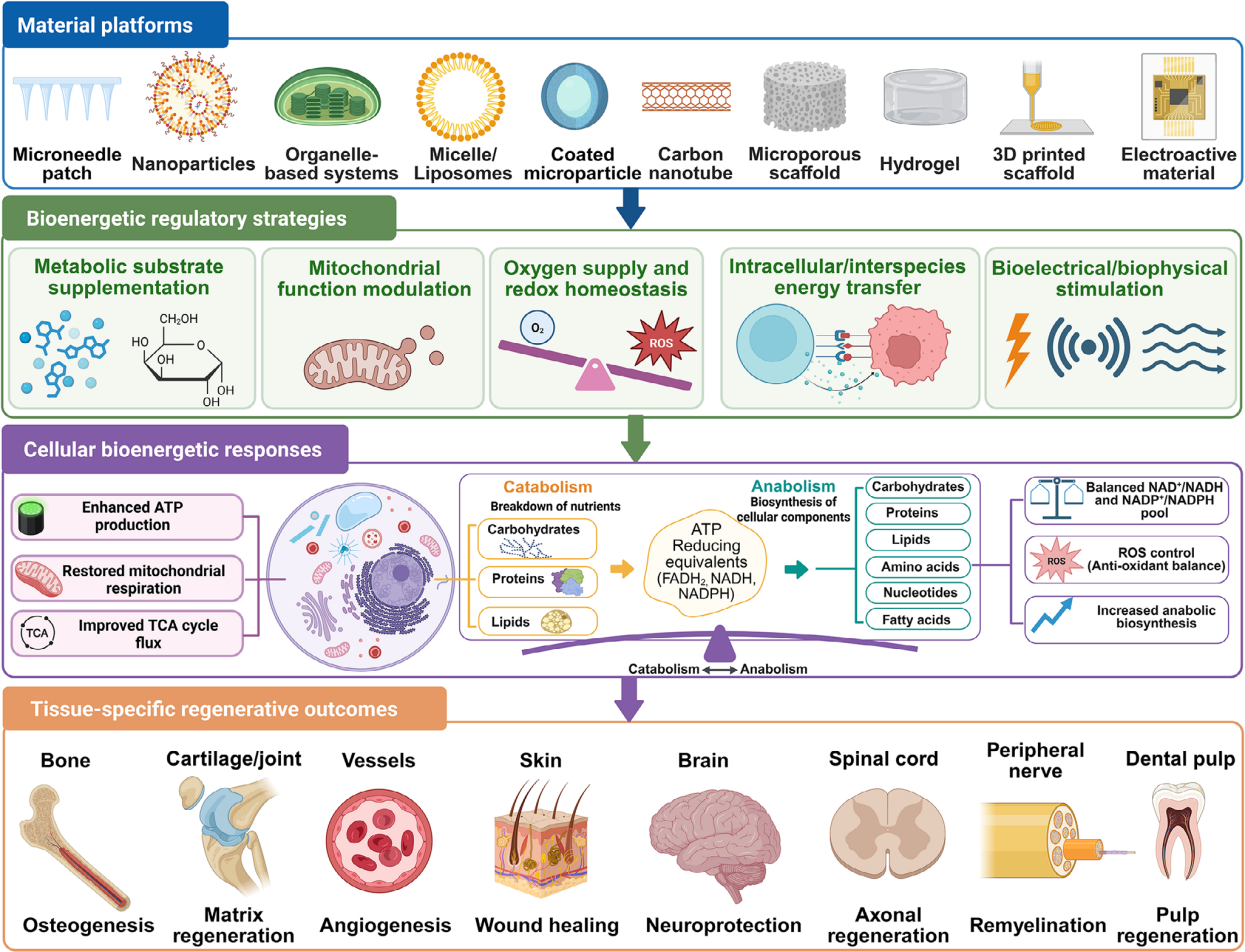
Bioenergetic materials promote tissue regeneration through regulation of cellular metabolism. A variety of material platforms, including microneedle patches, nanoparticles, organelles, micelles, coated microparticles, carbon nanotubes, microporous scaffolds, hydrogels, and 3D printed systems, can be engineered into bioenergetic materials. These platforms regulate cellular bioenergetics through several major strategies, including metabolic substrate supplementation, mitochondrial function modulation, oxygen supply, redox homeostasis restoration, intercellular or interspecies energy transfer, and bioelectrical or biophysical stimulation. Such interventions enhance ATP production, restore mitochondrial respiration, improve TCA cycle flux, maintain redox balance, and coordinate catabolic and anabolic metabolism by regulating reducing equivalents and biosynthetic pathways. The resulting restoration of cellular energy homeostasis supports tissue‐specific regenerative responses, including osteogenesis and angiogenesis in bone, matrix restoration in cartilage, vascular repair, wound healing, neuroprotection in the brain, axonal regeneration in the spinal cord, remyelination and functional recovery in peripheral nerves, and dental pulp regeneration. Abbreviations: ATP, adenosine triphosphate; FADH_2_, reduced flavin adenine dinucleotide; NAD^+^/NADH, oxidized/reduced nicotinamide adenine dinucleotide; NADP^+^/NADPH, oxidized/reduced nicotinamide adenine dinucleotide phosphate; O_2_, oxygen; ROS, reactive oxygen species; TCA, tricarboxylic acid (Created with BioRender.com).

Recent advances in BEMs have established the feasibility of metabolically targeted regenerative therapies. However, existing studies and reviews largely focus on individual material platforms or isolated metabolic mechanisms, with limited integration of the mechanistic relationships between biomaterial design, bioenergetic regulation, and tissue regeneration [20, 22, 23]. A unified framework that systematically links material engineering strategies to cellular bioenergetics remains lacking. In this review, we present a comprehensive synthesis of bioenergetic materials by integrating biomaterial design with the principles of cellular bioenergetics and metabolic regulation. We first introduce the concept of BEMs and their principal mechanisms of action. Then summarize their applications in bone, cartilage, skin, and neural tissue regeneration and conclude by discussing the major challenges and future directions for the clinical translation of bioenergetic therapies (Figure 1). By bridging metabolic biology with biomaterials engineering, this review aims to provide a conceptual framework for the rational design of next‐generation bioenergetic materials to enhance tissue regeneration.

## 2 Mechanisms of Action of Bioenergetic Materials

**FIGURE 2:**
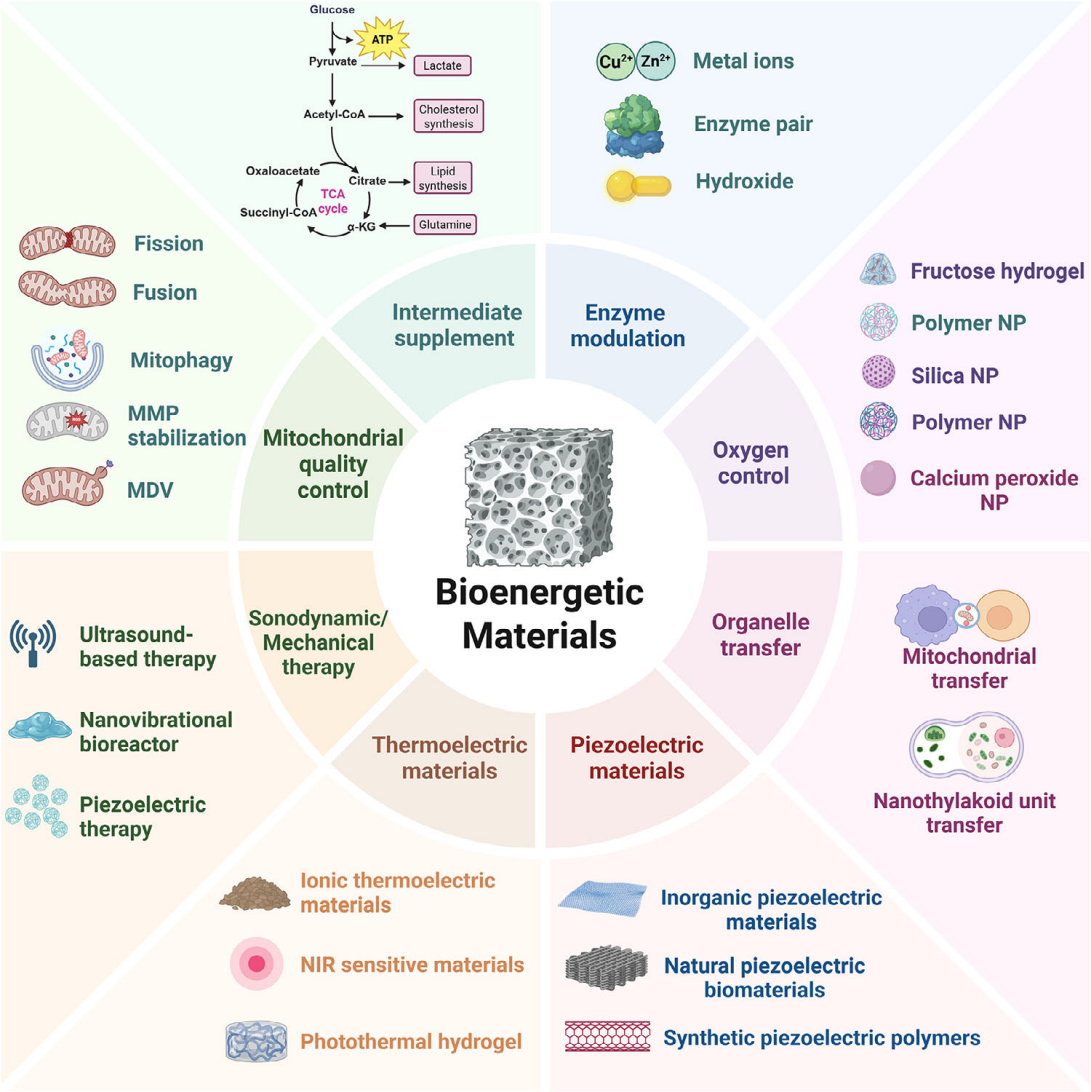
Mechanisms by which bioenergetic materials regulate metabolism and tissue regeneration. Bioenergetic materials regulate cellular metabolism and energy homeostasis through multiple complementary strategies. Metabolic intermediate supplementation supports glycolysis and the TCA cycle and provides substrates (e.g., glutamine and fatty acids) for ATP production, as well as precursors (e.g., lipids and amino acids) for biosynthetic pathways. Enzyme modulation uses catalytic components, such as metal ions, enzyme pairs, and hydroxide‐based materials, to regulate metabolic reactions and redox processes. Oxygen‐control systems, including fructose hydrogels, polymer nanoparticles, silica nanoparticles, and calcium peroxide‐based nanoparticles, modulate local oxygen availability and oxidative metabolism. Organelle‐transfer strategies restore cellular bioenergetics through mitochondrial transfer or the delivery of photosynthetic nanothylakoid units. Piezoelectric materials generate local electrical cues through mechanical deformation‐induced polarization, built‐in electric fields, and functional heterojunctions or metal‐piezoelectric nanostructures. Thermoelectric materials, conversely, convert thermal inputs into bioelectrical stimulation, and can be further integrated with photothermal components (e.g., NIR‐responsive materials or hydrogels) to enable light‐controlled thermal gradients for wireless stimulation. Sonodynamic and mechanical strategies, including nanovibrational bioreactors, ultrasound‐responsive hydrogels, and piezoelectric heterojunctions, transduce external energy inputs into biochemical signals that influence cellular metabolism and function. Mitochondrial quality‐control strategies further maintain bioenergetic homeostasis by regulating mitochondrial fission, fusion, mitophagy, mitochondrial membrane potential, and mitochondria‐derived vesicle formation. Collectively, these approaches illustrate how bioenergetic materials integrate metabolic, enzymatic, redox, organelle‐transfer, electrical, thermal, and mechanical cues to restore cellular energy balance and promote tissue regeneration. Abbreviations: ATP, adenosine triphosphate; α‐KG, alpha‐ketoglutarate; MDV, mitochondria‐derived vesicle; MMP, mitochondrial membrane potential; NIR, near‐infrared; NP, nanoparticle; TCA, tricarboxylic acid. (Created with BioRender.com).

Cellular bioenergetics (CBE) encompasses the fundamental networks of energy transformation, through which cells acquire, convert, store, and utilize energy to sustain cellular functions [24]. CBE is governed by an interconnected network comprising substrate availability, enzymatic flux, mitochondrial integrity, oxygen availability, redox balance, and electrochemical signaling [17, 25]. At the core of CBE lies the production of adenosine triphosphate (ATP), the universal energy currency required to support virtually all anabolic and regenerative processes [20, 24, 26]. BEMs represent a versatile class of engineered or naturally derived biomaterials designed to modulate CBE by targeting one or more components of these bioenergetic networks. These materials promote anabolic activity and tissue regeneration by enhancing, restoring, or reprogramming compromised cellular bioenergetic networks [24]. The principal mechanisms through which BEMs achieve bioenergetic modulation include directly supplying metabolic substrates or cofactors, modulating the catalytic ability of key metabolic enzymes, regulating ionic flux or local oxygen microenvironment, facilitating intercellular transfer of bioenergetic machinery such as mitochondria, and transducing electrical or mechanoelectrical cues that influence cellular energy metabolism [20]. (Figure 2 and Table 1)

**TABLE 1:** Bioenergetic strategies targeting metabolism for tissue regeneration.

Strategy	Example	Strength	Limitation	Ref.
Metabolic substrates supplementation	Polycaprolactone scaffolds containing α‐KG	Directly provides energy‐related metabolites to support mitochondrial metabolism and anabolic repair.	Excessive or unbalanced substrate supply may disturb metabolic flux and redox balance.	[168]
Metabolic enzyme modulation	galaxin‐GelMA	Enables more precise regulation of rate‐limiting metabolic pathways and influences cell fate decisions	Off‐target effects and toxicity are major concerns regarding the broad systemic functions of metabolic enzymes	[99]
Oxygen microenvironment control	CPO‐coated BCP	Restores oxidative phosphorylation by alleviating hypoxia and enhances cell survival, angiogenesis, and integration of engineered tissues	Risk of ROS overproduction and oxidative stress.	[169]
Organelle transfer	Gel@MDI	Can rescue severely damaged or metabolically compromised cells by directly restoring cellular bioenergetics.	Low delivery efficiency and potential immunogenicity and compatibility issues.	[66]
Mitochondrial homeostasis	Exo@hydrogel	Targets mitochondrial quality control processes, which are closely linked to stem cell function, aging, and regeneration capacity.	Limited biomaterial systems capable of precise mitochondrial targeting.	[104]
Piezoelectric regulation	TP‐hNG@GP‐IFP	Converts mechanical stimuli into electrical signals that can enhance mitochondrial activity and ATP production.	Dependence on external or endogenous mechanical stimulation and the electrical output is difficult to precisely control in vivo.	[170]
Thermoelectric regulation	BGS/MCFS	Converts temperature gradients into electrical signals, offering a self‐powered metabolic stimulation strategy.	Requires sufficient and stable temperature gradients, which are often weak in vivo.	[105]
Magnetoelectric regulation	PCLG/AgNF nerve guidance conduits	Enables non‐invasive and remote stimulation, especially for CNS and deep organs	Limited targeting specificity at cellular levels and dependent on external magnetic field systems	[79]
Triboelectric regulation	Ultrasound‐driven biodegradable vagus nerve stimulator	Provides self‐powered and sustainable bioelectrical stimulation with simple structure and flexible integration	Highly dependent on mechanical input with limited penetration depth.	[85]

Abbreviations: AgNF, silver nanofiber; ATP, adenosine triphosphate; BCP, biphasic calcium phosphate; BGS, Bi2S3‐based glass system; CNS, central nervous system; CPO, calcium peroxide; Exo, exosome; GelMA, gelatin methacryloyl; GP‐IFP, gold‐coated polymer internal fixation plate; MCFS, multifunctional composite fibrous scaffold; MDI, mitochondrial dynamics inhibitor; PCL, polycaprolactone; PCLG, gelatin‐modified polycaprolactone; ROS, reactive oxygen species; TP‐hNG, triboelectric/piezoelectric hybrid nanogenerator; α‐KG, alpha‐ketoglutarate.

### 2.1 Metabolic Substrate Supplementation

Metabolic substrate supplementation acts at the most upstream level of bioenergetic regulation by increasing the availability of carbon intermediates or energy‐relevant metabolites that can enter the TCA cycle [27]. Consequently, a primary class of BEMs is engineered to enhance intracellular bioenergetics by delivering canonical TCA cycle intermediates or closely associated metabolic precursors [24]. These canonical intermediates include citrate, isocitrate, α‐ketoglutarate (α‐KG), succinyl‐CoA, succinate, fumarate, malate, and oxaloacetate [28]. Beyond these direct cycle intermediates, however, numerous other molecules dictate cycle flux by supplying carbon backbones, serving as allosteric modulators, or driving parallel metabolic shunts [29]. These include entry‐point substrates such as pyruvate, [22] alternative fuels like lactate [30] and ketone bodies, [31] amino acid‐derived carbon sources (e.g., branched‐chain amino acid catabolites), and regulatory/signaling metabolites including itaconate, 2‐hydroxyglutarate, GABA, and lipid‐derived regulators [28, 32]. The main advantage of this strategy lies in its conceptual simplicity. The bioenergetic cue is built directly into the degradable matrix and can be released locally, without the need for exogenous cells or genetic modification. Its main limitations, however, include dependence on release kinetics, cellular uptake efficiency, and the metabolic competence of recipient cells. Free metabolites may also show poor permeability, dose constraints, or rapid hydrolysis, which prompts ongoing efforts to develop sustained‐release and cell‐permeable formulations.

BEMs developed to address these limitations typically consist of precisely tailored, biodegradable matrices that undergo controlled in situ degradation. This degradation locally releases small‐molecule fragments that are readily internalized by surrounding cells and funneled directly into the TCA cycle. For example, Yang and colleagues leveraged citrate to develop BEMs capable of driving metabonegenic regulation [33]. Specifically, they synthesized a highly photoluminescent biodegradable polymer (BPLP‐PSer) via a simple, one‐opt polycondensation of citrate, phosphoserine (PSer), and a diol. This polymer was subsequently fabricated into BPLP‐PSer/hydroxyapatite microparticulate scaffolds, which mimic the native bone extracellular matrix by presenting PSer‐enriched bioactive surfaces. Subsequent in vivo evaluations demonstrated that these composite scaffolds significantly accelerated bone regeneration in both rat femoral condyle and cranial defect models [33]. In addition to replenishing the TCA cycle and enhancing ATP generation, these intermediates may provide carbon skeletons for biosynthetic reactions and influence redox balance, epigenetic regulation, and cell differentiation in a context‐dependent manner. For example, α‐KG participates in dioxygenase‐dependent epigenetic regulation [34], succinate can affect redox and hypoxia‐related signaling [35], and citrate‐derived acetyl‐CoA contributes to lipid synthesis and histone acetylation [36, 37].

α‐KG is a critical intermediate in the TCA cycle and a potent endogenous anti‐aging molecule across various organisms [38]. Leveraging these dual properties, Sun and colleagues synthesized biodegradable α‐KG‐based polymeric microparticles (PAKG MPs) designed for sustained metabolite release [31]. Notably, these PAKG MPs exhibited exceptional cellular internalization by both pre‐osteoblasts and bone marrow mesenchymal stem cells (BMSCs), significantly outperforming the uptake efficiency of traditional polymeric carriers such as poly(L‐lactic acid) (PLLA) and poly(lactic‐co‐glycolic acid) (PLGA) [31]. Consequently, this PAKG platform serves as a highly effective BEM to simultaneously drive osteogenic differentiation and facilitate intracellular drug delivery [31]. In another application, Yu and collaborator developed a bioactive hydrogel incorporating spiky mesoporous silica nanoparticles (MSNs) loaded with α‐KG, while D‐mannose was covalently conjugated within the hydrogel network [39]. The sustained co‐release of α‐KG and D‐mannose effectively rescued the osteogenic differentiation of BMSCs and promoted the anti‐inflammatory polarization of macrophages (M2‐like) within a compromised diabetic microenvironment. Ultimately, this targeted metabolic intervention synergistically suppressed local inflammation and accelerated diabetic bone regeneration [39]. Sun and co‐workers used gelatin as a scaffold to release dimethyl α‐KG (DMAKG) at the bone defects. These DMAKG significantly promoted BMP2‐induced bone regeneration in aged mice via enhancing TCA cycle metabolism [40].

Succinate is a four‐carbon dicarboxylic acid and a key intermediate in the TCA cycle. Intracellularly, succinate is generated from succinyl‐CoA and subsequently converted to fumarate by succinate dehydrogenase (SDH), a process that drives mitochondrial ATP production by directly coupling to the electron transport chain [41]. Leveraging its bioenergetic potential, Zhang and colleagues formulated poly(glycerol succinate)‐based polymers to engineer a highly porous, biodegradable bioenergetic‐active material capable of sustained succinate release [20]. Upon degradation, these succinate‐donating fragments upregulated osteogenic gene expression (*Runx2*, *OCN*), enhanced alkaline phosphatase activity, and accelerated mineralized matrix deposition by host MSCs [20]. As a result, in a rabbit femoral defect model, these materials substantially improved bone volume, mineral density, and overall structural integrity [20]. Beyond osteogenesis, succinate is increasingly recognized for its immunoregulatory properties, positioning it as a valuable metabolic cure for cancer immunotherapy [42, 43]. Harnessing this mechanism, Acharya and colleagues incorporated succinate into a polymer backbone to create poly(ethylene succinate) (PES) microparticles [44]. This immunometabolic BEM was designed to reprogram the metabolism of innate phagocytic cells, shifting them toward a pro‐inflammatory phenotype [44]. This localized metabolic rewiring enhanced the activation of antigen‐presenting cells and expanded the population of RORγT‐expressing cytotoxic T cells (Tc17), culminating in effective, immune‐mediated tumor suppression [44]. These findings suggest that targeted immunometabolic modulation could be repurposed to bias macrophages toward pro‐regenerative phenotypes, enhance angiogenesis, and support tissue regeneration.

Malate is a crucial intermediate in the TCA cycle that bridges mitochondrial respiration with cytoplasmic glycolysis, acting as a primary component of the malate‐aspartate shuttle [45]. Recognizing that exogenous L‐malate can accelerate cellular bioenergetics and biosynthesis, Guo and colleagues developed a poly(diol L‐malate) (PDoM)‐based BEM scaffold. This platform was synthesized via a one‐pot polycondensation of L‐malic acid and aliphatic diols, followed by scaffold fabrication and thermal cross‐linking [46]. As the scaffold undergoes degradation, it locally releases L‐malate and active oligomeric fragments. Following cellular internalization, these substrates boost mitochondrial ATP production and reprogram the local metabolic microenvironment. This metabolic enhancement significantly accelerated tissue regeneration in a full‐thickness skin defect model [46]. Lactate forms when glucose is broken down in anaerobic glycolysis. It can be used as an energy source across various tissues via the lactate shuttle [47]. Beyond its role as a fundamental energy source, lactate also functions as a crucial immunometabolic regulator [48, 49]. Capitalizing on these properties, Tan and colleagues designed an anti‐inflammatory, lactate‐releasing BEM composed of waterborne polyurethane incorporated with poly‐L‐lactic acid (PLLA) to promote nerve regeneration in the absence of exogenous growth factors [50]. As the matrix degrades, the PLLA component governs the sustained release of lactate. In glucose‐deprived environments, local neurons import this lactate to drive mitochondrial ATP synthesis and fuel axonal outgrowth. When implanted into rat brain lesions, this BEM promoted filamentous neurite extension, rapid angiogenesis, and functional tissue regeneration over 28 days [50]. Furthermore, integrated metabolomic and transcriptomic analyses revealed that this regeneration was driven by the activation of purine metabolism pathways, linking neuroactive receptor‐ligand interactions with cAMP‐calcium signaling networks and angiogenic gene programs [50]. Expanding the utility of lactate to bone tissue engineering, Zhao and colleagues developed a three‐dimensional (3D) printed polycaprolactone/nano‐hydroxyapatite BEM scaffold functionalized with sodium lactate (SL) [30]. The sustained release of SL modulated the local metabolic microenvironment and activated metabolic‐epigenetic crosstalk, specifically promoting the osteogenesis of BMSCs via the lysine lactylation of STAT1 [30]. Subsequent in vivo evaluations confirmed that this BEM scaffold successfully repaired critical‐sized defects and promoted robust trabecular bone formation [30]. These two lactate‐based examples also highlight an important tissue‐dependent difference. In neural repair, lactate mainly serves as an alternative energetic substrate that supports mitochondrial ATP production and axonal growth under glucose‐limited conditions. In bone regeneration, however, lactate appears to act more broadly as a metabolic–epigenetic signal that regulates BMSC osteogenesis. Recent studies have shown that lactate can promote bone repair through multiple mechanisms, including endothelial cell‐derived histone lactylation, receptor‐mediated Olfr1440 signaling, exercise‐associated GPR81 activation, and scaffold‐mediated STAT1 lactylation. Thus, lactate‐based BEMs should not be viewed as uniformly pro‐regenerative additives. Their effects depend on delivery mode, local concentration, exposure duration, target cell type, and downstream effector pathway. This distinction is particularly important because pathological lactate accumulation under chronic hypoxic stress may impair BMSC osteogenesis and disturb lineage commitment. Therefore, the key advantage of scaffold‐mediated lactate delivery lies not simply in lactate supplementation per se, but in the ability to establish a localized and sustained pro‐regenerative lactate window at the defect site.

Taken together, substrate‐supplementing BEMs operate through distinct but overlapping bioenergetic mechanisms. The strength of metabolic evidence varies considerably among previous studies. Citrate‐, α‐KG‐, succinate‐, and malate‐based materials primarily increase the local availability of TCA cycle‐related substrates. By contrast, lactate can function either as an alternative energetic fuel or as a metabolic‐epigenetic signal depending on the tissue context. Importantly, however, direct evidence of enhanced cellular bioenergetics is not equally established across these platforms. Some studies directly linked substrate delivery to enhanced TCA cycle metabolism or mitochondrial ATP production [20, 33, 46, 50]. Others primarily demonstrated downstream outcomes, such as osteogenic differentiation, mineralization, immune modulation, angiogenesis, or tissue regeneration, without directly quantifying metabolic flux or energetic output [30, 31, 39, 40]. The biological consequences are also highly tissue‐dependent: substrate supplementation has predominantly been used to promote osteogenesis in bone defects [20, 30, 31, 33, 39, 40]. Malate and lactate have been exploited to support energy‐demanding repair in skin and neural tissues, respectively [46, 50]. Thus, the principal advantage of substrate‐supplementing BEMs is the ability to integrate metabolite delivery into degradable biomaterials for localized and sustained metabolic intervention. However, increasing extracellular substrate availability does not necessarily translate into predictable intracellular metabolic flux. Cellular uptake, release kinetics, baseline mitochondrial competence, local nutrient and oxygen availability, and the signaling properties of individual metabolites can all influence the regenerative outcome. Future studies should therefore complement histological and functional endpoints with direct measurements of ATP production, mitochondrial respiration, and metabolic flux to establish whether improved tissue regeneration is truly attributable to bioenergetic rescue. In addition, increasing substrate supply alone does not guarantee durable metabolic reprogramming, because intracellular flux also depends on catalytic control, cofactor handling, and local biochemical feedback. This leads naturally to enzyme‐ and ion‐mediated metabolic modulation.

### 2.2 Metabolic Enzyme Modulation

A second class of bioenergetic materials modulates metabolic enzymes, enzyme‐like catalysts, or ion‐dependent metabolic signaling, rather than simply supplying substrates. Encapsulating metabolism‐regulating enzymes within biomaterial scaffolds for controlled release is a powerful strategy for microenvironmental programming [51, 52]. For example, enhancing the activity of antioxidases is a promising strategy to treat various inflammation‐associated disorders [53]. Demonstrating this, Yan and colleagues presented a rationally designed artificial antioxidase BEM based on ruthenium‐doped layered double hydroxide (Ru‐hydroxide) to achieve effective redox regulation and promote maxillofacial bone repair [54]. Ru‐hydroxide incorporates single‐atom Ru centers coordinated with hydroxyl groups, forming a synergistic architecture capable of efficiently interacting with reactive oxygen species (ROS). This configuration facilitates rapid proton and electron transport, resulting in broad‐spectrum and potent ROS‐neutralizing activity [54]. Subsequent in vivo evaluations confirmed that this Ru‐hydroxide modulated the inflammatory milieu and supported osteogenic differentiation during bone regeneration in male murine models [54]. Expanding beyond purely local chemical cues, researchers are also engineering bioelectric BEM platforms to remotely control cellular metabolism. For instance, Kajimura and collaborator reported an implantable wireless optogenetic device which selectively stimulated adipocytes, enabling precise activation of Ca^2+^ cycling‐mediated thermogenesis [55]. The system integrates a compact power‐harvesting coil (2 mm in diameter), whose terminals are linked to a receiving circuit equipped with a rectifier. This configuration efficiently drives a blue micro‐LED, designed to genetically activate channelrhodopsin‐2 and actively modulate metabolic flux [55]. To ensure long‐term functionality, the device was encapsulated within a dual‐layer structure consisting of acrylic and Parylene‐C, providing both electrical insulation and resistance to biodegradation [55]. Mechanistically, optogenetic activation of channelrhodopsin‐2 induced Ca^2+^ influx and subsequent Ca^2+^ release from the endoplasmic reticulum via RyR2 (Ryanodine receptor 2) and IP3R (Inositol 1,4,5‐trisphosphate receptor), thereby driving ATP‐dependent Ca^2+^ cycling through the metabolic enzyme sarcoplasmic/endoplasmic reticulum Ca^2+^‐ATPase 2. This process markedly increased ATP hydrolysis, oxygen consumption, and glucose oxidation, highlighting a direct link between enzyme‐mediated Ca^2+^ cycling and metabolic reprogramming in adipocytes [55].

Inorganic ions are critical modulators of metabolic enzymes, frequently functioning as essential cofactors or allosteric activators that enhance the reactivity of biomacromolecules [56]. Beyond catalytic activation, inorganic ions can form coordination complexes with biological molecules, actively contributing to macromolecular architecture and cellular structure [57]. To harness these effects, Yan and colleagues designed a multifunctional BEM nanodepot composed of a bioactive core–shell CaF_2_‐based upconversion nanostructure, which co‐delivers multiple mineral ions to orchestrate and monitor biomineralization dynamics [58]. The controlled release of these ions directly promoted the nucleation and expansion of inorganic crystals while concurrently accelerating in vivo bone tissue repair [58]. In the context of bone regeneration, calcium phosphate (CaP)‐based BEMs undergo controlled degradation to yield calcium (Ca^2+^) and phosphate ions (PO_4_^3−^), both of which are critical regulators of local homeostasis [59]. Notably, the specific immunometabolic function of PO_4_^3−^ has recently been elucidated. Wu and colleagues demonstrated that PO_4_^3−^ ions released from CaP ceramics enter host macrophages via the SLC20a1 transporter. Once internalized, these ions boost ATP production through the TCA cycle and oxidative phosphorylation (OXPHOS). This bioenergetic surge downregulates AMPK, thereby activating mTOR signaling to drive the anti‐inflammatory M2 polarization of macrophages [59]. Furthermore, a portion of this intracellular ATP is secreted and metabolized into adenosine, which reinforces M2 polarization through A_2_B adenosine receptor signaling. Collectively, these findings establish inorganic ions as potent bioenergetic modulators capable of directly coupling immunometabolic regulation with tissue regeneration [59].

Taken together, enzyme‐ and ion‐modulating BEMs regulate cellular bioenergetics through mechanistically distinct routes, encompassing catalytic redox control, ATP‐consuming ion cycling, and ion‐mediated regulation of mitochondrial metabolism. The metabolic evidence supporting these strategies also differs in strength. Optogenetically induced Ca^2+^ cycling provided direct evidence of metabolic reprogramming, including increased ATP hydrolysis, oxygen consumption, and glucose oxidation [55]. CaP‐derived PO_4_^3−^ represents a more direct example in the tissue‐regenerative context, wherein enhanced TCA cycle activity and OXPHOS increased ATP production and subsequently regulated AMPK‐mTOR and adenosine signaling to promote macrophage M2 polarization and bone regeneration [59]. Thus, compared with simple substrate supplementation, enzyme‐ and ion‐based approaches can provide catalytic amplification or engage endogenous metabolic control nodes without relying solely on metabolite availability [54, 55, 59]. However, their effects are highly dependent on tissue context and local physicochemical conditions. Excessive catalytic activity, ion accumulation, or sustained perturbation of ATP‐consuming processes may disrupt metabolic homeostasis. Future studies should therefore define appropriate activity and concentration windows and incorporate direct measurements of metabolic flux, mitochondrial function, and energetic state alongside regenerative outcomes [60]. The efficacy of these enzyme‐ and ion‐mediated processes remains strongly constrained by local oxygen availability and redox conditions [60, 61], the next mechanistic layer involves direct regulation of oxygen tension and redox homeostasis [61].

### 2.3 Oxygen Tension and Redox Homeostasis Modulation

Oxygen tension and redox homeostasis are often decisive bottlenecks in ischemic, diabetic, infected, or thick engineered tissues. In these settings, tissue hypoxia greatly limits intracellular ATP production, severely hindering the process of wound repair and regeneration [62]. To overcome this limitation, Xie and colleagues designed a novel 3D‐bioprinted BEM scaffold featuring a coaxial design composed of gelatin methacrylate (GelMA) and alginate [63]. This biphasic system comprises a core layer embedded with calcium peroxide (CaO_2_) nanoparticles to ensure sustained oxygen generation, surrounded by an outer shell incorporating liposome‐encapsulated ATP to directly support cellular energy metabolism [63]. By finely tuning the rapid release of oxygen and the gradual diffusion of ATP, this BEM fosters improved cell viability and proliferation under hypoxic conditions, promoting efficient wound closure in a type II diabetic murine model [63].

Addressing the need for alternative energy substrates under ischemic conditions, Zhang and colleagues developed an injectable, high‐energy intermediate fructose hydrogel (HIFH) by dynamically coordinating Cu^2+^, sulfhydryl‐functionalized bovine serum albumin, and the high‐energy glycolytic intermediate fructose‐1,6‐bisphosphate [64]. Under in vitro hypoxic stress, this HIFH system effectively increased ATP production in endothelial cells by 1.3‐fold, thereby significantly rescuing cell viability, proliferation, antioxidant capacity, and migratory function. Furthermore, the BEM markedly promoted angiogenesis, evidenced by enhanced tube formation and the elevated secretion of pro‐angiogenic factors such as vascular endothelial growth factor (VEGF) and basic fibroblast growth factor (bFGF) [64]. Subsequent in vivo experiments using a rat ischemic skin model demonstrated that HIFH treatment accelerated tissue regeneration rates by approximately 1.4‐fold compared to untreated controls. These findings underscore the clinical potential of BEMs to bypass hypoxic metabolic bottlenecks via the direct delivery of high‐energy intermediates, ensuring robust tissue repair even in severely compromised microenvironments [64].

Exploiting the distinct metabolic vulnerabilities of bacterial cells compared to mammalian hosts, ROS generation has emerged as a potent antimicrobial strategy [65, 66]. Physiologically, immune cells combat bacterial invasion by diverting electrons from the mitochondrial electron transport chain to react with molecular oxygen, thereby generating targeted, lethal ROS bursts [67]. Wang and colleagues identified “mitoxyperiosis,” a distinct form of lytic cell death triggered by innate immune signaling and severe metabolic imbalance. In vivo, the activation of this ROS‐driven process successfully drove tumor regression in an mTORC2‐dependent manner [68]. To synthetically replicate and enhance these oxidative bursts, researchers are engineering photothermal and photocatalytic BEMs. Wu and colleagues, for instance, engineered a heterojunction BEM consisting of Ti_3_C_2_T*_x_*‐MXene and a (CoCrFeMnNi)_3_O_4_ high‐entropy oxide (HEO) [69]. Under near‐infrared (NIR) irradiation, the MXene generated photothermal “hot” electrons that were injected into the HEO surface. This dynamic electron redistribution facilitated rapid local oxygen adsorption and activation, yielding massive ROS production [69]. This engineered ROS release disrupted the bacterial electron transport chain, thereby halting ATP biosynthesis and compromising both the heat resistance and virulence of the pathogen [69].

Moving beyond light‐activated systems, BEMs can also be designed to physically extract electrons or harness mechanical energy. Employing a targeted approach, Dong and colleagues designed an iron‐copper bimetallic metal‐organic framework (MOF) with peroxidase‐like activity [70]. This BEM selectively bound to bacterial surfaces via boronic acid‐*cis*‐diol interactions to catalyze localized oxidative damage to bacteria. Crucially, the framework also facilitated directional electron extraction directly from the bacterial membrane, actively depleting the pathogen's oxidative phosphorylation capacity and ATP production [70]. Utilizing mechanical energy, Li and colleagues embedded barium titanate nanoparticles (BTO NPs) within a polycaprolactone (PCL) substrate to establish a piezoelectric BEM [71]. Upon non‐invasive ultrasound stimulation, the piezoelectric effect triggered localized electron release, inducing severe oxidative stress at the implant‐bacteria interface. This mechanically driven ROS generation eradicated adherent *Staphylococcus aureus* biofilms by irreversibly compromising membrane integrity and exhausting intracellular sugar‐based energy reserves [71].

To summarize, oxygen‐ and redox‐modulating BEMs operate through two fundamentally different bioenergetic modes: restoring energy metabolism in host cells or disrupting energy production in pathogenic cells. In hypoxic regenerative tissues, oxygen‐generating and energy‐supplementing platforms directly counteract energetic insufficiency to promote tissue repair. For example, CaO_2_/ATP scaffold that combined sustained oxygen generation with exogenous ATP delivery improved epithelial cell survival and proliferation under hypoxia [63]. HIFH provided more direct metabolic evidence, increasing endothelial ATP production by approximately 1.3‐fold under hypoxia and coupling this energetic rescue to improved endothelial function, angiogenesis, and tissue regeneration [64]. In contrast, antimicrobial redox‐active BEMs exploit the opposite bioenergetic principle. They generate ROS, redistribute or extract electrons, or harness mechanical energy to impair bacterial electron transport, oxidative phosphorylation, and intracellular energy reserves [69, 70, 71]. These strategies therefore contribute to tissue repair indirectly by removing an infection‐associated metabolic barrier rather than by directly enhancing host cell anabolism. Across both modes, a major advantage is their ability to manipulate oxygen‐ and electron‐dependent processes that become limiting in compromised microenvironments [62, 63, 64, 69, 70, 71]. However, their therapeutic window is intrinsically narrow because insufficient oxygen or ROS modulation may be ineffective, whereas excessive oxygen release, peroxide accumulation, or radical generation can damage host cells and aggravate oxidative stress. Moreover, several antimicrobial platforms rely on external NIR or ultrasound stimulation [69, 71], introducing additional constraints related to tissue penetration, scaffold geometry, and stimulus control. Future designs should therefore prioritize cell‐selective and spatiotemporally responsive regulation of oxygen and redox activity, together with direct measurements of ATP metabolism, oxygen consumption, redox state, and host‐pathogen selectivity. Responsive biomaterials hold significant promise for overcoming the constraints imposed by the narrow therapeutic window. For example, Liu and colleagues developed a multifunctional bilayer hydrogel system to achieve the precise release of bioactive components by responding to dynamic changes in wound ROS [72]. When restoring the extracellular microenvironment alone proves insufficient, bioenergetic materials can intervene more directly at the level of organelles and intracellular energy‐transducing machinery [73].

### 2.4 Mitochondrial Transfer & Photosynthetic Bioenergetic Systems

**FIGURE 3:**
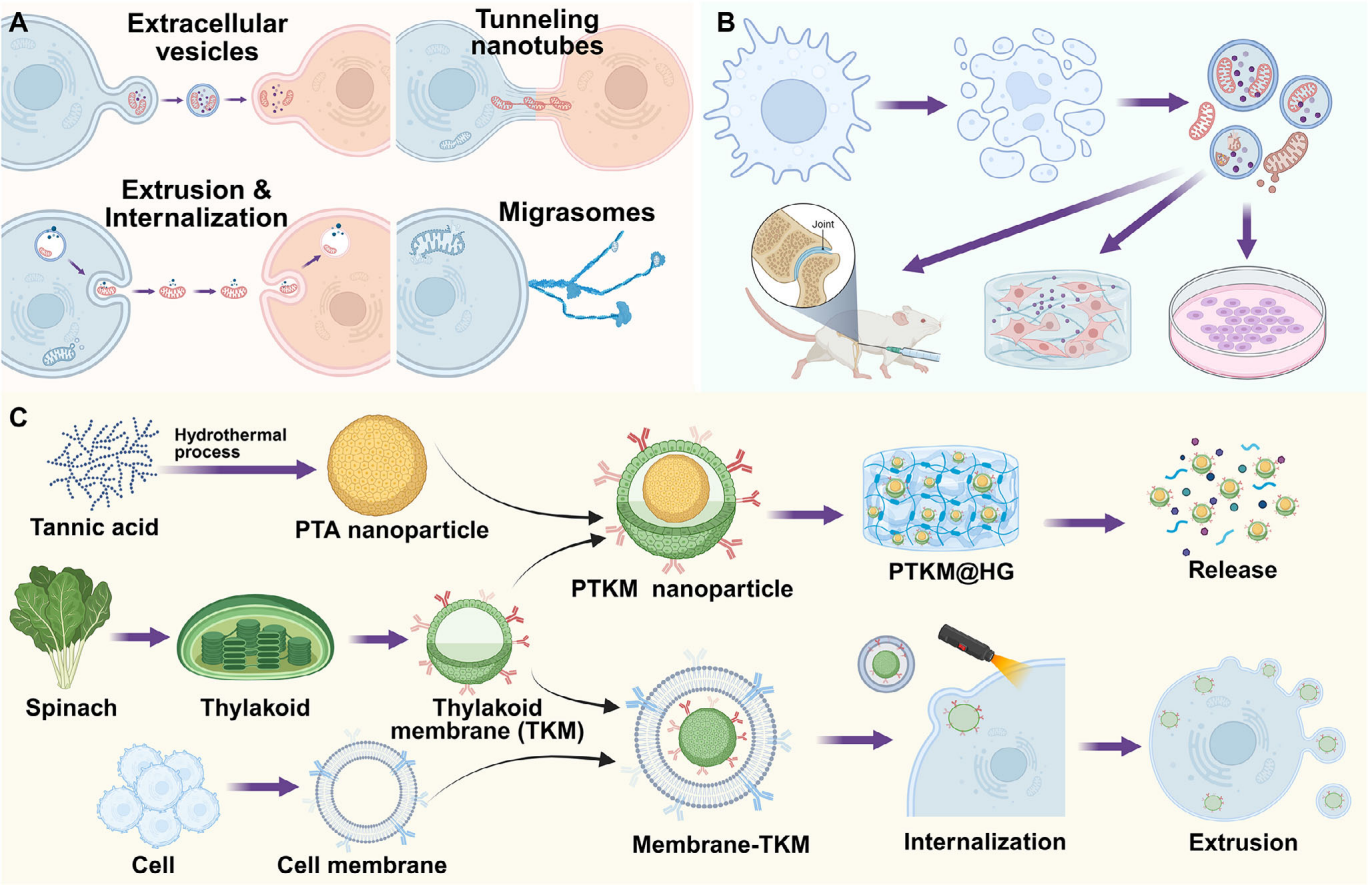
Strategies for organelle transfer and engineered nanostructure‐based delivery. (A) Natural organelle transfer occurs through extracellular vesicles (EVs), tunneling nanotubes (TNTs), extrusion and internalization mechanisms, and migrasomes. These processes allow the exchange of functional organelles, such as mitochondria, between donor and recipient cells. (B) Exogenous strategies for organelle transplantation involve the isolation of intact organelles and their delivery into target cells in vitro or in vivo via, for example, intra‐articular injection for tissue repair or incorporation into cell culture systems. (C) Engineered bioenergetic nanostructures integrate multiple biological and synthetic components. Tannic acid (TA) is processed hydrothermally into poly‐TA nanoparticles (PTA NPs), which are then coated with thylakoid membrane (TKM) to form PTKM NPs. These constructs are embedded in hydrogels (PTKM@HG), released in a sustained manner, internalized by recipient cells, and further disseminated through extrusion, enabling efficient metabolic support and regenerative function. Plant‐derived photosynthetic TKM can also be combined with cell membrane coatings to generate membrane‐TKM hybrids, which can be efficiently internalized by cells. (Created with BioRender.com).

Mitochondrial transfer and mitochondria‐targeting systems represent a more direct form of metabolic rescue, aimed at restoring the ATP‐producing machinery rather than merely adjusting extracellular supply conditions. Specially engineered BEMs can directly modulate mitochondrial function and even mediate the physical transportation of targeted organelles between discrete cell populations (Figure 3A,B) and across species (Figure 3C).

Showcasing targeted intracellular modulation, Zeng and colleagues designed a biomimetic BEM delivery system called HKL‐GECM@MPNPs to treat osteoarthritis (OA) [74]. It was constructed by encapsulating honokiol (HKL) within ROS‐responsive, mitochondria‐targeting polymer‐lipid hybrid nanocores (MPNPs), which were subsequently cloaked in genetically engineered chondrocyte membranes (GECM) that highly express IL‐1R2. This biomimetic design endowed the particles with prolonged joint cavity retention and deep cartilage penetration. Upon cytoplasmic entry, the nanocores homed to mitochondria, where they restored SIRT3 levels, increased ATP production by 1.14‐fold, enhanced oxidative respiration by 0.59‐fold, and suppressed pathological glycolysis by 0.45‐fold [74]. Furthermore, transmission electron microscopy confirmed that HKL‐GECM@MPNPs successfully reduced mitochondrial swelling, restored cristae architecture, and normalized overall organelle morphology [74]. Expanding from intracellular repair to intracellular energy sharing, Yang and colleagues developed a sophisticated hierarchical BEM hydrogel (Gel@MDI), incorporating macrophage‐targeted zwitterionic nanogels (MDV) within an injectable chitosan framework [75]. Utilizing reversible Schiff base chemistry, the hydrogel initially releases dimethyl itaconate to rapidly shift local macrophages toward a reparative M2 phenotype. Subsequently, the MDV nanogels deliver Miro1‐DNA directly to these macrophages. This targeted genetic reprogramming actively facilitates the physical transfer of healthy mitochondria from the M2 macrophages to adjacent BMSCs via tunneling nanotubes (TNTs) [75]. By functionally donating this bioenergetic machinery, Gel@MDI significantly restored mitochondrial respiration in recipient BMSCs, ultimately driving robust osteogenesis and resolving inflammation in a critical‐size mouse calvarial defect model [75].

Another highly innovative bioenergetic strategy leverages nature's photosynthetic machinery to augment ATP and NADPH production within injured tissues [76]. Utilizing the capacity of plant chloroplasts to generate abundant energy carriers during photosynthesis, Lin and colleagues extracted nanoscale vesicles from plant thylakoid membranes to create independent, controllable nanothylakoid units (NTUs) [77]. (Figure 3) To ensure immune evasion and targeted delivery, these NTUs were cloaked in mammalian chondrocyte cell membranes. Upon cellular internalization and light illumination, these NTUs autonomously produced ATP and NADPH in situ, directly augmenting the anabolic capacity of the host cells. In metabolically degenerated chondrocytes, this bioenergetic supplementation successfully increased extracellular matrix synthesis and restored metabolic homeostasis [77]. Similarly, Han and colleagues developed a visible‐light‐driven BEM hydrogel (PTKM@HG) incorporating thylakoid membrane‐encapsulated polyphenol nanoparticles (PTKM NPs) [78]. Under red light exposure, these embedded nanothylakoids execute oxygenic photosynthesis, simultaneously generating oxygen (O_2_) and scavenging excess ROS (H_2_O_2_) while yielding ATP and NADPH. This localized oxygen release relieves tissue hypoxia and improves mitochondrial respiration in ischemic wounds. Concurrently, the direct supply of ATP and NADPH fuels cellular anabolism and activates the leucine–mTOR signaling pathway, promoting diabetic wound healing [78]. In essence, these BEMs function as implantable artificial chloroplasts, microscopic power plants capable of converting external optical energy into usable biochemical fuel to drive mammalian tissue repair.

These strategies represent progressively more direct interventions in cellular bioenergetics, encompassing repairing endogenous mitochondria, transferring functional organelles, and introducing exogenous photosynthetic energy‐generating machinery. Mitochondria‐targeted nanomedicine offers the most conservative approach by restoring existing metabolic machinery. For example, HKL‐GECM@MPNPs increased ATP production and oxidative respiration in chondrocytes while suppressing pathological glycolysis, linking mitochondrial recovery to improved cartilage homeostasis [74]. In contrast, Gel@MDI bypasses intrinsic mitochondrial dysfunction by promoting the transfer of intact, functional mitochondria between cells, thereby restoring respiratory capacity in metabolically compromised recipient cells [75]. This approach may provide a broader bioenergetic rescue than modulation of a single mitochondrial pathway, but it relies on complex intercellular processes, including donor‐cell reprogramming, Miro1‐dependent mitochondrial trafficking, and tunneling nanotube formation [75], which may complicate mechanistic control and clinical translation. Photosynthetic systems extend this concept further by introducing an orthogonal energy‐conversion pathway that does not depend entirely on endogenous mitochondrial metabolism. NTUs and PTKM@HG can generate ATP and NADPH under illumination, while the latter additionally produces O_2_ and regulates ROS, thereby simultaneously addressing energetic insufficiency, hypoxia, and redox imbalance [77, 78]. However, this multifunctionality also makes it difficult to determine whether regenerative benefits arise primarily from direct ATP/NADPH supplementation, improved oxygenation, redox regulation, or secondary restoration of mitochondrial respiration. More importantly, photosynthetic BEMs remain constrained by the requirement for external light, limited penetration into deep tissues, and unresolved questions regarding the biodistribution, persistence, immunogenicity, and long‐term fate of plant‐derived bioenergetic components. Thus, although organelle‐ and photosynthesis‐based BEMs are among the most direct means of manipulating cellular energy production, their increasing bioenergetic autonomy is accompanied by increasing biological and translational complexity. Future studies should focus on these limitations and establish quantitative relationships among ATP/NADPH production, mitochondrial respiration, anabolic pathway activation, and functional tissue regeneration. Beyond biochemical and organelle‐based energy conversion, BEMs can also exploit electrical and electromechanical cues to regulate membrane potential, charge transfer, and energy‐dependent cellular functions, providing a conceptual bridge to electroactive bioenergetic materials [79].

### 2.5 Electrical Current Regulation

Electrical and mechanoelectrical BEMs regulate metabolism by converting mechanical, magnetic, optical, or triboelectric inputs into localized electrical outputs that influence membrane signaling, ion flux, and interfacial electron transfer [21, 80, 81]. Within BEM design, this is typically achieved through the incorporation of heterojunctions, interfaces formed between two distinct semiconductor materials. These heterojunctions exhibit unique electronic characteristics capable of inducing thermal, optical, and electrical phenomena often absent in natural biomaterials [82], presenting extensive applications across energy harvesting, optoelectronic sensing, and regenerative medicine [83, 84]. Building upon the photocatalytic heterojunctions discussed previously [69, 70], Mao and colleagues utilized the piezoelectric properties of Bi_2_WO_6_ combined with the semiconductor properties of TiO_2_ to create a built‐in electric field (IEF) at the heterojunction interface [85]. This bioelectronic BEM effectively integrated cell‐induced electrical stimulation with photodynamic and photothermal antibacterial therapies. Specifically, mechanical forces from surrounding cells activated the piezoelectric effect to generate continuous, localized electrical signals, which enhanced osteogenic differentiation. Near‐infrared irradiation (NIR) is a common external energy input that can be converted into localized thermal and redox effects, enabling thermoelectric‐mediated ROS generation and antibacterial activity against pathogens such as E. coli, methicillin‐resistant S. aureus (MRSA), and P. gingivalis [85].

In another innovative cross‐disciplinary approach, Deng and colleagues developed a living probiotic bio‐heterojunction (P‐bioHJ) BEM by integrating *Lactobacillus rhamnosus* with MXene (Ti3C2) quantum dots (MQDs) and an FeS heterojunction [86]. This dynamic BEM dismantled the dense extracellular polymeric substances (EPS) of established biofilms by disrupting bacterial metabolism, thereby enhancing deep antimicrobial penetration. Upon NIR, the P‐bioHJ simultaneously generated ROS and heat to completely eradicate the biofilm structure [86]. Beyond its primary antimicrobial action, the P‐bioHJ actively reduced local inflammation, promoted antioxidant pathways, and accelerated tissue regeneration, demonstrating therapeutic versatility in both in vitro and in vivo experiments [86].

Expanding on the utility of advanced nanomaterials for infection control, Dai and colleagues developed a photothermal BEM hydrogel incorporating a vancomycin‐loaded, quaternized chitosan‐coated bio‐metal‐organic framework (QCSMOF‐Van) [87]. This composite matrix was synthesized from GelMA and oxidized sodium alginate methacrylic acid (OSAMA) via dynamic Schiff base cross‐linking and electrostatic assembly [87]. During the in vivo healing process, the polycationic, positively charged QCS surface actively captures local bacteria, while the synergistic co‐release of broad‐spectrum Zn^2+^ ions and vancomycin potently suppresses bacterial metabolism to eradicate the infection [87]. Returning to the concept of IEFs, Yu and colleagues designed a three‐dimensional semiconductor heterojunction network (3D‐NTBH) implant using TiO_2_ nanowires and Bi_2_O_3_ nanodots [88]. The 3D heterojunction interfaces of this bioelectronic BEM facilitate rapid electron transfer and establish nanoscale internal electric fields. Spatially, this continuous electric field significantly accelerates glycolytic metabolism in surrounding endothelial cells, thereby meeting the bioenergetic demands required for early angiogenic activities [88]. Ultimately, this study demonstrates that modulating cellular energy metabolism via engineered semiconductor surfaces is a highly effective strategy to promote rapid vascular network reconstruction and implant integration [88]. Applying a similar dual‐action approach, Wu and colleagues modified Ti6Al4V orthopaedic implants with a hydroxyapatite/molybdenum sulfide (HA/MoS_2_) heterojunction coating via laser cladding and chemical vapor deposition (CVD) [89]. This advanced BEM interface was designed to facilitate directional electron transfer from the bacterial membrane to the implant surface. By exploiting the inherent metabolic energy differences between prokaryotes and mammalian cells, this continuous electron extraction selectively collapses bacterial metabolism [89]. Simultaneously, the engineered surface promotes osteogenesis in host MSCs by upregulating intracellular calcium signaling, showcasing the immense clinical potential of bioenergetic semiconductor implants [89].

Magnetoelectric and triboelectric materials are other energy‐conversion systems for transferring mechanical stimuli into bioelectrical signals. Magnetoelectric materials are designed to enable remote electrical stimulation through magnetic‐field‐induced electrical outputs. For example, Ren and co‐workers presented magneto‐electric coupling‐enabled electroactive nerve guidance conduits for wireless peripheral nerve regeneration. Mechanistically, magneto‐electric stimulation activates taurine and hypotaurine metabolism, increasing intracellular taurine levels to support neuronal proliferation, migration, and peripheral nerve repair [90]. Bu and colleagues developed a magneto‐electronic strategy to drive cancer cells into a senescent state. The enhanced catalytic hydrogenation reaction of FePt‐FeC heterostructures under alternating magnetic stimulation decreased the concentration of an essential coenzyme‐β‐nicotinamide adenine dinucleotide‐in cancer cells. This metabolic vulnerability was further exploited by enhanced Fenton‐like reactions for effective tumor cell killing [91]. Surmenev and colleagues developed a magnetoelectric nanoplatform for targeted delivery to the central nervous system (CNS) by leveraging axonal transport pathways. Core–shell MnFe_2_O_4_@Ba_0_._85_Ca_0_._15_Zr_0_._1_Ti_0_._9_O_3_ nanoparticles (MFO@BCZT) were used as nanotransducers that convert low‐intensity alternating magnetic fields into localized electrical stimulation. This activation enhances neuronal calcium signaling and promotes cellular uptake and axonal transport of cargoes from the nasal cavity to the brain [92]. Liu and collaborators developed dopamine‐modified Fe_3_O_4_@BaTiO_3_ magnetoelectric nanoparticles, which enables wireless stimulation‐induced CXCL12 upregulation and autophagy activation, activating bioelectronic‐metabolic regulatory axis for neural regeneration [93]. Triboelectric systems generate bioelectric signals via contact electrification and mechanical motion, providing self‐powered platforms for cellular regulation. Mohammadpour and colleagues developed an on‐demand antibacterial platform based on triboelectric nanogenerator (TENG)‐induced electrical stimulation of Cu_2_S substrates. TENG‐generated currents captured electrons from bacterial electron transport chains, achieving antibacterial effects by inducing oxidative stress and metabolic dysfunction [94]. TENG‐generated pulses have also been applied to tumor therapy by increasing ATP leakage and high mobility group box 1 protein release in tumor cells [95]. Zhang and co‐workers developed a fully biodegradable, ultrasound‐powered vagus nerve stimulator (UBVS) for wireless neuromodulation in murine models. The device integrates a triboelectric energy harvester with a self‐adherent neural interface, enabling stable, suture‐free chronic stimulation of the vagus nerve. Functionally, UBVS reprogrammed the neuroimmune‐metabolic axis by suppressing inflammation and remodeling lipid metabolism, leading to reduced atherosclerotic plaque burden [96].

Taken together, electroactive BEMs provide a distinct route to bioenergetic regulation by converting endogenous or externally applied physical energy into electrical or interfacial electronic cues. However, the mechanistic connection to cellular bioenergetics varies substantially across current platforms. Semiconductor heterojunctions provide the clearest host‐directed metabolic evidence when their built‐in electric fields directly alter cellular energy metabolism [88]. In contrast, semiconductor heterojunction [89] and triboelectric systems [94] primarily act on bacterial bioenergetics by extracting electrons from bacterial electron transport chains, disrupting metabolic activity, or inducing oxidative stress [89, 94]. These platforms therefore facilitate regeneration mainly by controlling the metabolism of bacteria. The major strength of electroactive BEMs lies in their capacity for spatially and temporally controllable regulation without continuous delivery of metabolic substrates. Their major limitations include material complexity, strong dependence of biological responses on stimulation intensity, frequency, and duration, and the lack of rigorous long‐term safety and dosing studies, particularly when antibacterial redox chemistry and regenerative signaling are combined on the same interface [97]. Future studies should therefore distinguish electrical signaling effects from bona fide bioenergetic modulation by quantitatively linking electrical outputs to ATP production, mitochondrial respiration, glycolytic flux, or other direct energetic readouts, while also establishing long‐term safety and reproducible stimulation windows.

In summary, these five mechanistic categories represent distinct intervention points along a shared bioenergetic axis rather than competing design philosophies. Substrate‐releasing systems directly increase the availability of metabolic fuels but remain dependent on cellular uptake and intrinsic metabolic competence. Enzyme‐ and ion‐modulating materials provide catalytic amplification and access to endogenous metabolic control nodes, although their bioenergetic effects are often highly context‐ and concentration‐dependent. Oxygen‐ and redox‐regulating systems are particularly relevant to hypoxic, inflammatory, and infected tissues, but require careful control to balance energetic rescue against oxidative injury. Mitochondrial transfer and photosynthetic systems intervene most directly when endogenous energy‐producing machinery is compromised, yet this mechanistic potency comes at the cost of greater biological and translational complexity. Electroactive platforms offer externally or mechanically controllable regulation with high spatiotemporal precision, although direct evidence linking electrical cues to cellular energy metabolism remains heterogeneous across previous studies. Thus, no single strategy is universally optimal. The appropriate BEM design should be matched to the dominant bioenergetic constraint of the target tissue and supported by direct metabolic measurements.

## 3 Bioenergetic Materials Targeting Metabolic Reprogramming in Tissue Regeneration

Effective tissue regeneration requires host cells to enter a highly anabolic state. Consequently, BEMs have garnered attention for their unique capacity to reprogram intracellular metabolic networks rather than relying solely on traditional growth factors [20, 24, 98]. To better describe this process, Yang and colleagues recently proposed the term metabotissugenesis, defined as a coordinated regenerative process wherein metabolic regulation is intricately coupled with tissue‐specific signaling networks to drive the formation, repair, and functional integration of complex tissue systems [32, 98]. This framework demands a systems‐level perspective on tissue engineering, wherein localized metabolic substrates serve simultaneously as bioenergetic fuels and instructive cues for tissue repair. Translating this concept into practice, Yang and colleagues engineered CitraBoneQMg, a multifunctional citrate‐based BEM scaffold synthesized via a straightforward one‐pot incorporation of citrate, glutamine, and magnesium [32]. Beyond exhibiting unique photoluminescent and photoacoustic tracking capabilities, CitraBoneQMg provided a sustained, localized release of its bioactive constituents. Subsequent evaluations demonstrated that this controlled delivery enhanced mitochondrial bioenergetics, paradoxically co‐activating both the mTORC1 and AMPK pathways to drive efficient osteogenic differentiation in MSCs [32]. Building upon these foundational principles, this chapter explores the practical translation of BEMs across diverse clinical contexts, illustrating how the diverse mechanistic strategies discussed previously are specifically tailored to achieve functional regeneration in distinct target tissues.

### 3.1 Bioenergetic Therapy for Bone

While bone tissue possesses a strong intrinsic capacity for self‐repair, the management of critical‐sized bone defects remains a formidable challenge. This complex healing process requires not only the de novo formation of mineralized tissue but also robust angiogenesis, proper mechanical alignment, and functional integration with surrounding tissues [99]. Compounding this challenge, the intrinsic regenerative capacity of bone declines substantially with age [100, 101, 102]. To enhance bone regeneration, researchers have developed multiple therapeutic strategies, including advanced biomaterial scaffolds, bioactive molecule supplementation, cell and gene therapies, and biophysical stimulation [103]. Among these, BEMs have emerged as a highly promising paradigm. As discussed previously, current BEM approaches promote osteogenesis by supplementing TCA cycle intermediates [30, 31, 33], modulating key metabolic enzymes [59], facilitating intercellular organelle transfer [75], and harnessing biophysical cues such as piezoelectricity [85]. Crucially, successful bone repair requires the coordinated action of multiple distinct cell populations, including MSCs, osteoblasts, osteoclasts, endothelial cells, osteocytes, immune cells, and nerve cells [103]. In the following subsections and in Table 2, we detail how precisely engineered BEMs are utilized to target and reprogram different cells to drive comprehensive bone regeneration.

**TABLE 2:** Bioenergetic strategies targeting metabolism for bone regeneration.

Strategy	Target	Example	Effects	Mechanism	Ref.
Metabolic substrates supplementation	hMSC	BPLP‐PSer/HA microparticles	Promoted bone regeneration and tissue response in rat femoral‐condyle and cranial‐defect models.	Citrate uptake via SLC13a5 enhanced energy metabolism; citrate+PSer provided metabolic stimulation and imaging capability	[28]
	Pre‐osteoblasts and BMSC	PAKG MPs	Promoted osteoblastic differentiation, enhanced bone regeneration, and enabled efficient intracellular drug delivery.	Sustained α‐KG release restored metabolic activity, and activated Wnt/β‐catenin and PI3K‐Akt pathways	[30]
	BMSC	PCL/nHA/SL	Promoted adhesion, proliferation, osteogenic differentiation, and bone regeneration in critical‐sized defects.	Lactates induced lysine lactylation of STAT1, releasing RUNX2 and reprogramming metabolic–epigenetic osteogenesis	[29]
	Macrophages	BCP ceramics	Strong osteoinductivity; reduced long‐term inflammation	Phase composition directed macrophage polarization, shifting immune‐metabolic environment toward pro‐osteogenic (M2) states	[53]
	hAdMSCs	Polycaprolactone scaffolds containing α‐KG	Enhanced mineralized tissue formation, improved ECM organization, and restored osteogenic potential in T2D‐impaired cells.	α‐KG replenished mitochondrial metabolism, restored energy and amino acid balance under diabetic conditions.	[168]
	Macrophages	Citrate‐functionalized scaffold	Inhibited M1 polarization, promoted M2 polarization, maintained bone homeostasis, and provided a therapeutic strategy for osteoporosis.	Citrate blocked glycolysis‐related enzymes, redirected flux into the TCA cycle, and promoted OXPHOS to favor M2 anti‐inflammatory phenotype.	[172]
Metabolic enzyme modulation	BMSCs	SASS scaffolds	Promoted osteogenic differentiation of BMSCs, balanced bone resorption and bone formation, and enhanced bone regeneration in critical‐sized defects	Induced the expression of mitochondrial complex IV subunit 4 isoform 2 and enriched focal adhesion and osteogenic differentiation pathways in MSCs	[93]
	Aged MSCs	EM‐eNMs	Rejuvenated aged BMMSCs, restored stemness and osteogenic potential, and reversed osteoporotic bone loss in vivo.	Bound ATP synthase, induced mitophagy, mitochondrial fission, and glycolysis via DRP1 activation	[96]
	Pre‐osteoblasts	Galaxin‐GelMA	Promoted osteogenesis and enhanced bone regeneration in a mandibular defect model.	Interacted with the β subunit of ATP synthase, regulating mitochondrial metabolism and thereby boosting bioenergetic activity to drive osteogenic differentiation.	[99]
	Macrophages	Se‐MBG	Improved mitochondrial function and promoted bone regeneration in critical‐sized defects.	Se‐MBG scavenged excess intracellular ROS by upregulating GPX4, changing macrophage metabolism toward oxidative phosphorylation to shift them into a pro‐healing M2 phenotype	[107]
Oxygen control	Osteogenic lineage cells	CPO‐coated BCP	Enhanced new bone formation, upregulated osteogenic marker expression, and increased mineralization and biomechanical strength	Released oxygen, alleviating local hypoxia in the defect site. Restored oxygen supply prevented cell apoptosis and necrosis, supported mitochondrial respiration and oxidative metabolism	[169]
	hPDCs	PFO‐HPs	Prolonged cell survival under hypoxia, preserved osteogenic differentiation, and accelerated new bone formation with higher bone density.	Controlled release of oxygen from PFO‐HPs alleviated hypoxia, sustained mitochondrial respiration, and oxidative metabolism	[175]
	Osteoblasts osteoclasts	Strontium peroxide (SrO_2_)‐loaded poly(lactic‐co‐glycolic acid) (PLGA)/gelatin scaffold	Enhanced osteoblast proliferation, inhibited osteoclast formation, improved oxygenation, and promoted osteogenesis for bone defect repair.	Sr^2+^ release stimulated osteoblast activity and suppressed osteoclastogenesis. SrO_2_ decomposition produced O_2_, increasing local oxygen tension to support mitochondrial oxidative metabolism and energy production required for bone formation.	[176]
	MSCs	PFTBA	Enhanced MSC survival, increased osteocalcin activity, boosted ectopic and orthotopic bone formation, and improved bone quality and volume	PFTBA acted as a synthetic oxygen carrier, enriching oxygen supply to implanted MSCs	[177]
Organelle transfer	MSC	Gel@MDI	Promoted the regeneration of critical size calvarial defects	Reestablished metabolic balance by mediating mitochondrial transfer from macrophages to BMSCs	[66]
	Osteoprogenitors	MDVs	Promoted osteogenic differentiation of progenitors and enhanced bone regeneration in vivo	MDVs transferred from osteoblasts to osteoprogenitors acted as metabolic messengers to stimulate osteogenesis.	[98]
	Macrophages	Silicified collagen scaffold combined with a Drp1‐Fis1 interaction inhibitor	Restored vascularization, enhanced innervation, and promoted mineralized bone regeneration in diabetic bone defects.	Silicon regulated mitochondrial fission dynamics in macrophages via the Drp1‐Mff pathway. Functional mitochondria were transferred through microvesicles to endothelial and neuronal cells	[111]
Mitochondrial homeostasis	Endothelial cells	Exo@hydrogel	Repaired alveolar bone defect and promoted angiogenesis in diabetic rats	Promoted mitochondrial fusion and restored mitochondrial function in endothelial cells by upregulating the expression of Opa1.	[104]
Piezoelectric regulation	MSCs	TiO_2_/Bi_2_WO_6_ heterojunction	Eradicated bacteria and promoted osteogenesis and osteointegration	Under NIR light, heterojunctions generated ROS and heat; Mechanical forces triggered the heterojunctions to generate local electric fields	[74]
	HUVECs	PMPNs@ECM/nHAW	Promoted angiogenesis and osteogenesis, enhanced cell migration, scavenged ROS, improved biocompatibility, and accelerated bone tissue repair.	NIR enhanced metabolic activity driving vascularization and osteogenesis. Sr ion + EGCG release provided sustained chemical cues for osteogenic differentiation and angiogenesis.	[178]
	Osteoblasts	TP‐hNG@GP‐IFP	Promoted osteogenic differentiation, bone formation, and mineralization; accelerated bone defect repair through biofeedback electrical stimulation.	Bioelectric signals generated by heartbeat and respiratory motion reprogramed glucose metabolism. Metabolic shift to aerobic glycolysis activated osteogenesis‐related pathways.	[170]
	Macrophages	BaTiO_3_‐modified Ti6Al4V scaffold	Promoted M2 macrophage polarization, reduced inflammation, enhanced immunoregulatory osteogenesis, and improved bone regeneration	Inhibited MAPK/JNK inflammatory signaling, while activating OXPHOS and boosting ATP synthesis in macrophages.	[154]
Thermoelectric regulation	Osteoblasts and endothelial cells	BGS/MCFS	Eliminated periprosthetic infections and promoted osteogenesis and angiogenesis	Antibacterial cations and the near‐infrared‐II photothermal property inhibited bacterial energy and material metabolism	[105]
	Macrophages	MXene@PDA	Provided biocompatible scaffold support, promoted osteogenesis, modulated immune response, scavenged ROS, and eliminated bacteria under NIR stimulation.	NIR‐triggered photothermal stimulation from MXene@PDA regulated macrophage metabolism by reducing ROS‐induced inflammatory stress and promoting M2 polarization.	[180]
Mechanical regulation	MSCs	Nanovibrational bioreactor	Promoted osteoinduction and bone regeneration without chemical inducers	Vibrational amplitude stimulated energetic metabolic pathways	[95]
Sonodynamic therapy	Macrophages	Ultrasound‐responsive nanofiber hydrogel	Accelerated BMSC osteogenic differentiation and improved bone regeneration.	Promoted M2 macrophage differentiation, enhanced secretion of BMP‐2 and IGF‐I, activated glycolysis and the TCA cycle, and reduced ROS production	[108]
	Osteoblasts	DTO/BTO heterojunction scaffold	Achieved 99.82% bacterial eradication, inhibited biofilm formation, enhanced mitochondrial fusion, promoted osteoblastic differentiation, and supported bone defect repair.	Ultrasonic piezoelectric charges generated microcurrents to stimulate osteoblasts, enhancing mitochondrial fusion and metabolic activity.	[181]

Abbreviations: ATP, adenosine triphosphate; BCP, biphasic calcium phosphate; BMMSC, bone marrow mesenchymal stem/stromal cell; BMP‐2, bone morphogenetic protein 2; BMSC, bone marrow‐derived mesenchymal stem cell; DRP1, dynamin‐related protein 1; ECM, extracellular matrix; EGCG, epigallocatechin gallate; Exo, exosome; Fis1, mitochondrial fission 1 protein; GelMA, gelatin methacryloyl; GPX4, glutathione peroxidase 4; hAdMSC, human adipose‐derived mesenchymal stem cell; hMSC, human mesenchymal stem cell; hPDC, human periosteum‐derived cell; HUVEC, human umbilical vein endothelial cell; IGF‐I, insulin‐like growth factor I; JNK, c‐Jun N‐terminal kinase; MAPK, mitogen‐activated protein kinase; MDV, mitochondria‐derived vesicle; Mff, mitochondrial fission factor; MP, microparticle; MSC, mesenchymal stem/stromal cell; nHA, nano‐hydroxyapatite; NIR, near‐infrared; OPA1, optic atrophy protein 1; OXPHOS, oxidative phosphorylation; PDA, polydopamine; PFTBA, perfluorotributylamine; PI3K–Akt, phosphoinositide 3‐kinase–protein kinase B signaling pathway; PLGA, poly(lactic‐co‐glycolic acid); PSer, phosphoserine; ROS, reactive oxygen species; RUNX2, runt‐related transcription factor 2; Se‐MBG, selenium‐containing mesoporous bioactive glass; SLC13A5, solute carrier family 13 member 5; STAT1, signal transducer and activator of transcription 1; T2D, type 2 diabetes; TCA, tricarboxylic acid cycle; α‐KG, alpha‐ketoglutarate.

Formulation abbreviations: BPLP‐PSer/HA, biodegradable photoluminescent polymer–phosphoserine/hydroxyapatite composite; EM‐eNMs, energy metabolism‐engaged nanomedicines; Exo@hydrogel, exosome‐loaded hydrogel; PAKG MPs, poly(alpha‐ketoglutarate) microparticles; PCL/nHA/SL, polycaprolactone/nano‐hydroxyapatite/sodium lactate composite; TP‐hNG, triboelectric/piezoelectric hybrid nanogenerator; GP‐IFP, gold‐coated polymer internal fixation plate; MXene@PDA, polydopamine‐modified MXene composite.

Chemical compounds and material components: BaTiO_3_, barium titanate; Bi_2_WO_6_, bismuth tungstate; O_2_, oxygen; Sr^2+^, strontium ion; SrO_2_, strontium peroxide; TiO_2_, titanium dioxide; Ti6Al4V, titanium–6 aluminum–4 vanadium alloy.

MSCs serve as the primary cellular reservoir for bone regeneration, not only by differentiating into osteoblasts but also by secreting cytokines and exosomes that modulate the healing process [104, 105, 106]. Consequently, bioenergetic strategies targeting MSCs have shown promising results in repairing bone defects. As discussed previously, the transfer of mitochondria from macrophages to MSCs is an effective method for boosting MSC metabolism and thereby enhancing osteogenesis [75]. Another strategy focuses on mechanically upregulating intrinsic mitochondrial function. Yang and colleagues developed deformed scaffolds incorporating graphite, fullerene, and diamond to provide gradient stress stimulation [106]. This scaffold architecture‐induced stress stimulation (SASS) upregulated the expression of mitochondrial complex IV subunit 4 isoform 2 (*Cox4i2*) in MSCs, driving the enhanced energy metabolism required for bone regeneration [106]. Building on these mechanically driven approaches, Dalby and colleagues engineered a nanovibrational bioreactor capable of modulating MSC metabolism [107]. Remarkably, this bioreactor induced MSC differentiation into osteoblasts in 3D culture without requiring specific osteogenic media or chemical cues [107]. At a 90 nm amplitude, the stimulated MSCs exhibited elevated levels of glycolytic and TCA cycle metabolites. Endowed with this mechanical memory, these MSCs, demonstrated superior osteogenic ability when subsequently embedded in tissue‐engineered scaffolds [108]. Beyond mechanical stimulation, restoring the stemness and osteogenic potential of aged bone marrow‐derived MSCs (BMMSCs) is an important strategy for reversing osteoporotic bone loss. Addressing this, Liu and colleagues utilized black phosphorus as a precursor to construct energy metabolism‐engaged nanomedicines (EM‐eNMs) via a contact‐electro‐catalysis approach [109]. These EM‐eNMs selectively targeted mitochondria of aged BMMSCs, directly binding to ATP synthase to drive mitochondrial fission, mitophagy, and glycolysis. This targeted metabolic reprogramming effectively rejuvenated the aged BMMSCs, enhancing their stemness and restoring their osteogenic differentiation capacity. In vivo, the systemic administration of EM‐eNMs in aged mice significantly mitigated osteoporotic bone loss and improved bone regeneration, highlighting a powerful bioenergetic strategy for treating age‐related bone degeneration [109].

Osteoblasts are the primary cells responsible for bone formation. They produce essential bone matrix components, such as type I collagen, osteopontin, and osteocalcin, while also secreting alkaline phosphatase to facilitate mineralization [110]. Derived from MSCs, these cells interact cooperatively to construct osteons, the fundamental structural units of bone [110]. Recent evidence highlights the importance of bioenergetic communication in this cellular network. For example, Lee and colleagues found that mature osteoblasts secrete mitochondria and mitochondrial‐derived vesicles (MDVs) into the extracellular matrix. These mitochondrial fragments are subsequently internalized by nearby osteoprogenitors, driving their differentiation and enhancing osteogenesis in vivo [111]. Targeting osteoblast metabolism directly, Zhao and colleagues used recombinant coral‐derived galaxin to develop a bioinspired gelatin methacryloyl (galaxin‐GelMA) scaffold [112]. By interacting directly with the β subunit of ATP synthase in osteoblasts, this scaffold significantly increased basal and ATP‐linked oxygen consumption rates, mitochondrial membrane potential, and overall ATP production, while simultaneously reducing intracellular and mitochondrial ROS levels. Subsequent in vivo evaluations confirmed that the galaxin‐GelMA scaffold successfully promoted bone regeneration in a murine model of penetrating mandibular defects [112].

Osteoclasts are specialized, bone‐resorbing cells derived from the hematopoietic lineage [113]. They are responsible for breaking down old or damaged tissue, a catabolic process tightly coupled to osteoblast‐mediated bone formation. This controlled resorptive activity is essential for remodeling newly formed bone into a mature, structurally resilient architecture [113]. Demonstrating the targeted bioenergetic modulation of this lineage, Shao and colleagues synthesized an electrolytic nanographene oxide (ENGO) hydrogel (ENGO/GelMA) [114]. This scaffold successfully enhanced type H vessel angiogenesis and subsequent bone regeneration by simultaneously stimulating preosteoclast‐derived PDGF‐BB secretion and inhibiting terminal osteoclast maturation. Mechanistically, ENGO exerted these regulatory effects by directly inhibiting isocitrate dehydrogenase 1 (IDH1), thereby suppressing local α‐KG production. This reduction in α‐KG inactivated the histone demethylase KDM7A, which in turn increased H3K9me2 methylation at the CTSK promoter, ultimately driving the epigenetic repression of osteoclast resorptive function [114].

Angiogenesis is intricately coupled with osteogenesis, a process that requires the migration and maturation of endothelial cells [115]. These cells form new capillaries that invade the healing bone, delivering essential oxygen, nutrients, and stem cells to the defect site [115]. Concurrently, VEGF released during the healing process not only drives this angiogenesis but also directly stimulates MSCs to undergo osteogenic differentiation [116]. Consequently, establishing a robust vascular network is a prerequisite for the successful healing of large bone defects. To enhance endothelial cell function and promote angiogenesis within a compromised hyperglycemic environment, Zhen and colleagues developed an injectable thermosensitive hydrogel loaded with MSC‐derived exosomes [117]. Upon delivery, these exosomes promoted mitochondrial fusion and restored overall mitochondrial bioenergetics in endothelial cells by upregulating the optic atrophy (Opa1) protein [117]. In another approach, Weng and colleagues designed a multifunctional bioactive glass scaffold (BGS) engineered via the topological transformation of polymetallic sulfide nanosheets rich in Mg^2+^, Cu^2+^, and Fe^3+^ [118]. The sustained release of these antibacterial cations successfully inhibited bacterial energy production and nutrient metabolism, thereby eliminating periprosthetic infections. Furthermore, the localized, sustained delivery of Mg^2+^ and Cu^2+^ from the nanosheets enhanced the intrinsic metabolism and angiogenic capacity of human umbilical vein endothelial cells (HUVECs), ultimately driving robust vascularized bone regeneration in a rabbit calvarial defect model [118].

Immune cells, particularly macrophages, are integral to both host defense and the orchestration of tissue regeneration. Specifically, the alternatively activated M2 macrophage phenotype is critical for facilitating bone healing, as it establishes an anti‐inflammatory, pro‐regenerative microenvironment conducive to osteogenic differentiation and tissue repair [119]. To actively regulate macrophage metabolic states, Lei and colleagues developed a responsive microsphere scaffold (D‐lip/CM@GM) via a microfluidic platform, combining gelatin methacrylate (GM) and carrageenan methacrylate (CM) [120]. This scaffold provided the sustained release of dimethyl fumarate (D‐lip), which was readily internalized by inflammatory macrophages. Intracellularly, D‐lip inhibited GAPDH, effectively blocking pathological glycolysis and restoring mitochondrial respiration. This metabolic shift suppressed NF‐κB activation and reduced the expression of proinflammatory mediators such as iNOS, IL‐1β, IL‐6, and TNF‐α, thereby driving macrophage polarization toward an anti‐inflammatory Arg‐1^+^ phenotype. Furthermore, by simultaneously targeting GAPDH, the scaffold eradicated planktonic, biofilm, and intracellular forms of MRSA. Ultimately, this dual‐action intervention controlled both infection and inflammation, metabolically reprogramming the microenvironment to promote tissue repair and bone regeneration [120]. In another approach targeting redox homeostasis, Guo and colleagues synthesized selenium‐doped mesoporous bioactive glass (Se‐MBG) [121]. This material upregulated the expression of the peroxide enzyme glutathione peroxidase 4 in macrophages, which enhanced oxidative phosphorylation and promoted M2 polarization to support robust bone repair [121]. Leveraging biophysical triggers, Sun and colleagues designed an ultrasound‐responsive nanofiber hydrogel containing a peptide equipped with M2‐regulatory and self‐assembly modules [122]. Upon ultrasound stimulation, the hydrogel gradually released these nanofibers, which boosted cellular glycolysis and the TCA cycle while concurrently suppressing ROS generation. This targeted metabolic modulation successfully promoted M2 macrophage polarization [122]. By inducing these M2 macrophages to secrete pro‐osteogenic factors like BMP‐2 and IGF‐I, the BEM system facilitated the osteogenic differentiation of BMSCs and significantly accelerated bone regeneration [122].

Nerve cells are pivotal regulators in the bone healing process [123]. Neuropeptides released from nerve fibers can influence both osteogenesis and angiogenesis, highlighting the close interaction among neural, vascular, immune, and bone compartments during tissue regeneration [124]. To improve neurovascularization in diabetic bone defects, Niu and colleagues developed a silicon‐modified collagen scaffold combined with a Drp1‐Fis1 interaction inhibitor to fine‐tune mitochondrial fission in macrophages [125]. Engineered by integrating bioactive silicon into a collagen matrix, this scaffold facilitates the controlled release of silicic acid at the defect site. This targeted bioenergetic stimulation enhanced overall mitochondrial function and upregulated the fission process within host macrophages, prompting them to package and deliver healthy mitochondria via microvesicles directly to endothelial and neuronal cells [125]. This intercellular mitochondrial transfer restored mitochondrial activity in recipient cells and supported the recovery of vascular and neural functions. In a diabetic mouse model of critical‐sized calvarial defects, the combined strategy enhanced vascularization and nerve regeneration and ultimately promoted new bone formation. These findings demonstrate a sophisticated biomaterial approach that elegantly couples macrophage metabolic regulation with neurovascular protection to drive bone regeneration [125].

Collectively, current BEM‐based strategies for bone regeneration coordinate metabolic regulation across multiple cell types, including MSCs, osteoblasts, osteoclasts, endothelial cells, macrophages, and nerve‐associated cells [126, 127]. These studies indicate that successful bone healing benefits from metabolic regulation that spans multiple cell compartments, rather than targeting osteoblasts alone. However, several limitations remain. For example, long‐term degradation safety, batch‐to‐batch reproducibility, sterilization compatibility, and performance in metabolically compromised settings remain insufficiently resolved. Another important consideration is the influence of the local mechanical environment. Because bone repair occurs under tissue‐specific mechanical stress, dynamic loading may substantially affect scaffold degradation, ion or metabolite release, cell metabolism, angiogenesis, and mineral deposition [115]. Current BEM studies evaluate regeneration under relatively static or simplified defect conditions, and the interaction between bioenergetic regulation and dynamic mechanical loading has not been systematically investigated. At the same time, the evidence remains mostly preclinical and frequently relies on rodent or rabbit defect models with modest follow‐up. It is still difficult to isolate the specific contribution of bioenergetic regulation from overlapping osteoconductive, ionic, mechanical, and immune effects. Future work should prioritize standardized metabolic endpoints, disease‐relevant large‐animal models, and head‐to‐head comparisons against clinically used bone substitutes.

### 3.2 Cartilage Repair and OA

Cartilage possesses a low intrinsic healing capacity, primarily due to the quiescent nature of resident chondrocytes and the avascular microenvironment of the tissue [128]. Under pathological conditions, these chondrocytes become metabolically impaired, compromising their ability to synthesize and maintain the cartilage matrix [129, 130]. Consequently, modulating cellular energy metabolism has emerged as an effective strategy for driving cartilage repair [131, 132]. Current bioenergetic approaches include mitochondrial transfer [133], chloroplast‐derived vesicle delivery [77], the enhancement of respiratory enzymes [134], and the direct supplementation of metabolic substrates [23]. While chondrocytes remain the primary focus of these interventions, other intra‐articular cell populations, such as macrophages, adipocytes, and synoviocytes, are increasingly recognized as therapeutic targets. Table 3 summarizes the diverse biomaterial strategies currently employed to reprogram metabolism for cartilage regeneration.

**TABLE 3:** Bioenergetic strategies targeting metabolism for cartilage regeneration.

**Strategy**	**Target**	**Example**	**Effects**	**Mechanism**	**Ref**.
Metabolic substrate supplementation	Chondrocyte	BAH	Enhanced ECM synthesis, restored mitochondrial function under oxidative stress, and accelerated cartilage repair in rabbit defect models.	Increased mitochondrial membrane potential and ATP production, reduced ROS, improved mitochondrial morphology by supplying metabolic intermediates	[20]
	Chondrocyte	Rh Gel@SP‐EVs	Eased pain, increased ECM deposition, lowered IL‐6 and OARSI scores, and normalized subchondral bone remodeling in OA mice	Re‐energized chondrocyte metabolism and improved cartilage repair via cutting ROS, restoring mitochondrial membrane potential, and raising ATP synthase	[118]
Metabolic enzyme modulation	Chondrocyte	Suc‐EXO	Filled defects quickly and integrated smoothly by forming hyaline‐like cartilage with higher ICRS scores and lower matrix metalloproteinase in rabbit cartilage defect models	Raised ATP and mitochondrial membrane potential of chondrocytes by elevating the mRNA level of TCA enzymes and mitochondrial fission and fusion related genes	[21]
	Chondrocytes Macrophages	Mn‐AKT‐MPs / Co‐AKT‐MPs	Scavenged ROS, protected ECM, and prevented cartilage degeneration. Suppressed synovial inflammation and painful symptoms in OA mice.	Multi‐enzyme mimetic activity allowed broad ROS elimination. Maintained balance of chondrocyte anabolism and catabolism by reducing oxidative stress.	[182]
Organelle transfer	Chondrocyte	MitoT	Restored ATP production, improved mitochondrial dynamics, reduced ROS and apoptosis, protected cartilage, and improved bone/joint repair in OA models.	Donated mitochondria boost OXPHOS, suppressed p‐DRP1, and upregulated MFN2 and SOD2 to enhance fusion, rebalance redox metabolism, and rescue energy dysfunction.	[115]
	Chondrocyte	CM‐NTUs	Boosted intracellular ATP and NADPH, restored anabolism, improved cartilage homeostasis, and protected against OA progression.	CM‐NTUs directly generated ATP and NADPH inside chondrocytes, restoring bioenergetic and redox balance, enhancing anabolic metabolism, and maintaining cartilage function.	[68]
Mitochondrial homeostasis	Macrophage	MHTCK	Achieved prolonged joint retention, alleviated pain, and protected cartilage in OA rats	Scavenged ROS, preserved ATP production, and promoted M2 macrophage polarization	[130]
	Chondrocyte	CTNM@FU	Reversed impaired cartilage metabolism, protected against cartilage degeneration, and promoted new cartilage formation in vivo.	Enabled delivery of fucoidan to chondrocytes, activated SIRT3, enhanced mitochondrial energy metabolism, and restored metabolic homeostasis.	[120]
Thermoelectric regulation	Chondrocytes	Mn_3_O_4_@PDA@Pd‐SS31 nanozymes	Scavenged mitochondrial ROS, restored mitochondrial function, promoted mitophagy, inhibited inflammation, and regenerated cartilage	NIR irradiation triggered release of Pd and Mn_3_O_4_; provided synergistic SOD‐ and CAT‐like enzyme activity, reducing mROS overload; restored mitochondrial function by balancing redox homeostasis and promoting mitophagy.	[184]
Others	Macrophage	LCF‐CSBN	Reduced inflammation, oxidative stress, and cartilage degeneration in OA rat model	Reprogramed macrophage lipid metabolism to promote efficient M1‐to‐M2 repolarization	[129]
	MSCs	Hydrogel‐LNP	Reduced synovial inflammation, restored cartilage structure, and promoted the differentiation of new chondrocytes	Recruited endogenous SMSCs and guided their differentiation toward chondrocytes; siRNA regulated cholesterol metabolism genes	[131]

Abbreviations: ATP, adenosine triphosphate; CAT, catalase; ECM, extracellular matrix; EV, extracellular vesicle; EXO, exosome; ICRS, International Cartilage Repair Society; IL‐6, interleukin 6; LNP, lipid nanoparticle; M2, alternatively activated macrophage phenotype; MFN2, mitofusin 2; mROS, mitochondrial reactive oxygen species; MSC, mesenchymal stem/stromal cell; NADPH, reduced nicotinamide adenine dinucleotide phosphate; NIR, near‐infrared; NP, nanoparticle; OA, osteoarthritis; OARSI, Osteoarthritis Research Society International; OXPHOS, oxidative phosphorylation; p‐DRP1, phosphorylated dynamin‐related protein 1; ROS, reactive oxygen species; siRNA, small interfering RNA; SIRT3, sirtuin 3; SMSC, synovial mesenchymal stem/stromal cell; SOD, superoxide dismutase; SOD2, superoxide dismutase 2; TCA, tricarboxylic acid cycle.

Chemical compounds and material components: Co, cobalt; Mn_3_O_4_, manganese(II, III) oxide; Pd, palladium; PDA, polydopamine; SS31, Szeto–Schiller peptide 31.

Formulation abbreviations: CM‐NTUs, cell membrane‐coated nanothylakoid units; Exo, exosomes; Hydrogel‐LNP, hydrogel‐integrated lipid nanoparticle system; MitoT, mitochondrial transplantation; Suc‐EXO, succinate‐modified exosomes; Mn_3_O_4_@PDA@Pd‐SS31, Mn_3_O_4_/polydopamine/palladium nanozyme functionalized with the mitochondria‐targeting peptide SS31.

Chondrocytes are the primary cellular targets for chondrogenesis. As discussed previously, NTUs cloaked in mature chondrocyte membranes were able to enter host chondrocytes through membrane fusion, increasing intracellular ATP and NADPH levels to improve anabolism in degenerated chondrocytes. This bioenergetic supplementation successfully corrected energy imbalances and resisted the pathological progression of OA [77]. Similarly, MSC‐derived mitochondria can be taken up by chondrocytes to promote metabolism and chondrogenesis, achieved either via co‐culture with MSCs or direct joint cavity injection [133].

Beyond direct mitochondrial modification, supplying intermediate metabolites or altering epigenetic states are effective methods for metabolism regulation. For example, Zhang and colleagues synthesized a prepolymer using glycerol, succinic acid, and itaconic acid, which was subsequently crosslinked under ultraviolet light to form a bioenergetic‐active hydrogel (BAH) [22]. This hydrogel exhibited excellent viscoelasticity, mechanical strength, and controlled degradability. During the degradation process, BAH continuously released succinic acid while maintaining overall structural stability. Importantly, BAH enhanced cellular bioenergetics by increasing the mitochondrial membrane potential and intracellular ATP levels of chondrocytes, thereby improving mitochondrial respiratory function and reducing the extracellular acidification rate. In a hydrogen peroxide (H_2_O_2_)‐induced oxidative stress model, BAH significantly reduced ROS accumulation, restored mitochondrial morphology and function, improved cell viability, and promoted ECM production [22]. In a rabbit cartilage defect model, BAH implantation demonstrated remarkable regenerative potential, promoting the formation of tissue that closely resembled native cartilage by 12 weeks [22]. Targeting genetic regulation, Mao and colleagues developed a chondrocyte‐targeted, phagocyte‐evading nanocarrier by decorating polyamidoamine with PEG, minimal “self” peptides, and chondrocyte‐affinity peptides [135]. This nanocarrier system, termed PMC, was loaded with mt‐tRF3b‐LeuTAA to generate PMC‐mt‐tRF3b‐LeuTAA. The resulting nano‐system penetrated human cartilage, preferentially entered chondrocytes, and exhibited selective uptake [135]. By delivering mt‐tRF3b‐LeuTAA, the system modulated chondrocyte metabolism and mitophagy, effectively restoring anabolism and mitochondrial function. In destabilization of the medial meniscus (DMM) mice, this nano‐system reduced osteophyte formation, attenuated cartilage degeneration, and preserved tissue integrity with minimal in vivo toxicity [135].

Extracellular vesicles (EVs), particularly exosomes, are also emerging as versatile nanocarriers for cartilage repair. Zhang and colleagues developed bioenergetic‐active exosomes (Suc‐EXO) by stimulating BMSCs with succinate [23]. The resulting Suc‐EXOs were enriched in TCA cycle intermediates and carried ∼5‐fold higher ATP levels than control EVs. When incorporated into an injectable hydrogel, Suc‐EXOs were released in a sustained manner and internalized by chondrocytes. The Suc‐EXOs raised endogenous ATP levels and mitochondrial membrane potential by upregulating TCA cycle enzymes and genes involved in mitochondrial dynamics [23]. In a rabbit defect model, the hydrogels more efficiently filled defects, achieving smooth integration by 6 weeks and forming hyaline‐like cartilage by 12 weeks, evidenced by high International Cartilage Repair Society (ICRS) scores and lower matrix metalloproteinase levels [23]. Similarly, Zhou and colleagues loaded *Spirulina*‐derived EVs (SP‐EVs) into a self‐assembled rhein hydrogel (Rh Gel@SP‐EVs) to enhance cartilage bioenergetics [136]. The 3D nanofiber network gradually released antioxidative and metabolic compounds [136]. Rh Gel@SP‐EVs re‐energized chondrocyte metabolism and improved cartilage repair by reducing ROS, restoring mitochondrial membrane potential, and upregulating ATP synthase. In DMM and monosodium iodoacetate (MIA)‐induced OA mice, the hydrogel eased pain, increased ECM deposition, lowered IL‐6 levels, and normalized subchondral bone remodeling [136]. Finally, Dong and colleagues used a strategy based on a natural compound. Catalpol, an iridoid glycoside, has been shown to promote chondrocyte anabolism and inhibit catabolic oxidative stress by suppressing heat shock protein 90β (Hsp90β) [137]. To optimize delivery, Dong and coworkers developed biodegradable mesoporous silica nanoparticle (bMSN) to load catalpol (Ca‐bMSN). This engineered system enabled the sustained release of catalpol, facilitated its penetration into chondrocytes, and effectively inhibited OA progression [137]. Additional bioenergetic strategies targeting chondrocytes are summarized in Table 3 [138, 139, 140].

Synoviocytes play essential roles in driving synovial inflammation, a key pathological feature of OA and cartilage degeneration [141]. Specifically, fibroblast‐like synoviocytes (FLSs) are intimately involved in propagating this inflammatory cascade. Targeting this pathway, Damerau and colleagues demonstrated that inhibiting pyruvate dehydrogenase kinases (PDKs) effectively shifted cellular metabolism in FLSs from pathological glycolysis back to oxidative phosphorylation, thereby suppressing FLS proliferation and decreasing inflammatory cytokine release [141]. Furthermore, Jiang and colleagues demonstrated that FLS‐derived exosomes (inf‐exo) induced synovitis and exacerbated OA progression by promoting macrophage M1 polarization via enhanced glycolysis. In a murine DMM model, intra‐articular injection of inf‐exo for 4–8 weeks led to aggravated cartilage degradation, increased matrix metalloproteinase 13 expression, and pronounced synovial inflammation compared with control exosomes or saline, accompanied by elevated F4/80^+^iNOS^+^ M1 macrophage infiltration [142]. To explore the therapeutic potential of metabolic reprogramming in synoviocytes, Zhao and colleagues synthesized a biomimetic, multifunctional metal‐organic framework nanoplatform (mZPMG NPs) [143]. This system was constructed by encapsulating methotrexate (MTX) and glucose oxidase (GOx) within a zinc imidazole framework‐8 (ZIF‐8), which was further functionalized with platinum nanoparticles (Pt NPs) and camouflaged using a macrophage membrane. By simultaneously inhibiting the abnormal proliferation of FLSs through GOx‐mediated starvation therapy and promoting macrophage polarization toward an M2 phenotype, the mZPMG NPs offered a synergistic strategy to suppress synovial hyperplasia and alleviate joint inflammation [143]. Similarly, Lee and colleagues developed ROS‐responsive, sulfasalazine (SSZ)‐loaded ferrocene nanoparticles to regulate lactate dehydrogenase activity in FLSs [144]. Evaluated in a 3D synovial hyperplasia model derived from patient FLSs, the platform effectively reduced intracellular ROS levels, suppressed lactate dehydrogenase activity, and downregulated inflammatory cytokine expression. Subsequent in vivo studies utilizing a collagen‐induced arthritis model confirmed these significant therapeutic effects, demonstrating marked cytokine modulation and a reduction in joint inflammation [144].

Low‐grade inflammation is a hallmark of OA and is closely associated with an imbalance between M1 and M2 synovial macrophages. Repolarizing pro‐inflammatory M1 macrophages toward the anti‐inflammatory M2 phenotype has emerged as a promising strategy to mitigate OA progression [145]. To enhance this conversion efficiency, Jiang and colleagues developed a camouflaged meta‐defensome. These macrophage‐membrane‐coated nanoparticles, co‐loaded with S‐methylisothiourea and MnO_2_, selectively targeted activated macrophages and accumulated in their mitochondria to restore aerobic respiration. By simultaneously scavenging mitochondrial ROS and inhibiting nitric oxide synthase, the system achieved an 82.3% repolarization efficiency [146]. In a collagenase‐induced OA model, the intravenous delivery of these meta‐defensomes markedly reduced synovial inflammation and slowed disease progression [146]. Targeting alternative metabolic pathways, Zeng and colleagues designed a self‐assembling licofelone‐loaded nanoparticle (LCF‐CSBN) constructed from chondroitin sulfate and bilirubin crosslinked via an ethylenediamine linker [147]. Exhibiting prolonged joint retention and selective accumulation in M1 macrophages, this nanoplatform delivered licofelone to selectively reprogram macrophage lipid metabolism. This intervention promoted M1‐to‐M2 repolarization, markedly reducing inflammation, oxidative stress, and cartilage degeneration in OA rat models [147]. Finally, directly regulating mitochondrial function is another effective method to promote M2 macrophage polarization. To achieve this, Zhou and colleagues developed a biomimetic nanoplatform (MHTCK) by integrating CeO_2_ nanozymes and the anti‐inflammatory peptide KAFAK within a macrophage‐synoviocyte membrane coating [148]. Upon targeting the mitochondria, MHTCK efficiently scavenged ROS, preserved ATP production, and promoted M2 macrophage polarization. In vivo evaluations demonstrated that MHTCK achieved prolonged joint retention for up to 10 days, significantly alleviating pain and protecting cartilage in OA rats [148].

Inducing the chondrogenic differentiation of stem cells represents another important strategy to enhance cartilage repair. Demonstrating this approach, Li and colleagues engineered an injectable hydrogel composed of self‐assembling peptide nanofibers designed to actively recruit endogenous synovial mesenchymal stem cells (SMSCs) and guide their differentiation into chondrocytes [149]. To complete this cellular recruitment, lipid nanoparticles (LNPs) carrying siRNA designed to regulate cholesterol metabolism were incorporated into the hydrogel, enabling their gradual release within the joint cavity. This dual‐function platform simultaneously improved the local inflammatory microenvironment, suppressed catabolic signaling, and enhanced the regenerative potential of the recruited stem cells [149]. In a rat model of OA induced by anterior cruciate ligament transection, intra‐articular administration of the hydrogel‐LNP system significantly alleviated synovial inflammation, restored overall cartilage architecture, and promoted the de novo differentiation of chondrocytes via enhancing MSC recruitment from the synovium and supporting their differentiation toward a chondrogenic lineage [149].

Taken together, BEMs offer promising opportunities for cartilage repair by improving chondrocyte mitochondrial function, restoring ATP production, reducing oxidative stress, and reshaping the inflammatory joint microenvironment. These effects have been achieved through diverse approaches, including photosynthetic nanothylakoid systems, mitochondria‐ or exosome‐mediated metabolic support, and nanoparticle platforms that restore mitophagy or redirect glycolytic drift. However, it is important to distinguish focal cartilage defects from OA when tailoring BEM‐based strategies. Unlike OA, focal defects are typically acute, isolated lesions in the presence of intact surrounding cartilage, synovium, and subchondral bone [150]. In this context, the local environment remains relatively healthy, and repair requires boosting energy availability within the naturally hypoxic cartilage matrix [151]. By contrast, OA is marked by an imbalance in macrophage phenotypes: pro‐inflammatory M1 macrophages, which rely on glycolysis, accumulate in the synovium, whereas anti‐inflammatory M2 macrophages depend on oxidative phosphorylation [152]. The resulting proinflammatory milieu in OA inhibits chondrogenesis and drives cartilage catabolism. Although focal defects exhibit lower baseline inflammation and reduced catabolic cytokine levels, they still face the challenge of repairing a dense, avascular matrix [153].

Thus, BEM design should be tailored to the distinct pathological features discussed above. In focal defects, BEMs may primarily focus on energy supplementation and structural support to empower chondrocytes for matrix reconstruction. In OA, however, BEMs must additionally act as immunometabolic regulators. Given that macrophage bioenergetics vary markedly by phenotype, these phenotype‐specific metabolic profiles should inform the rational design of BEM targets. A key strength of BEMs lies in their broad exploratory scope, as current studies have investigated multiple cellular targets and different mechanistic pathways. However, cartilage remains a difficult target for bioenergetic intervention because of its avascular structure, dense extracellular matrix, low cellularity, and limited intrinsic repair capacity. Thus, effective delivery of BEMs often necessitates mineralized or vascularized scaffolds, or combination with pro‐angiogenic approaches to facilitate chondrocyte access. In addition, many existing studies rely on chemically or surgically induced small‐animal OA models, which do not fully replicate the chronic, heterogeneous nature of human OA. Additional factors, including joint residence time, feasibility of repeat dosing, immunogenicity of complex biologic carriers, and long‐term effects on synovium and subchondral bone, also warrant further exploration.

### 3.3 Skin Wound Healing

**FIGURE 4:**
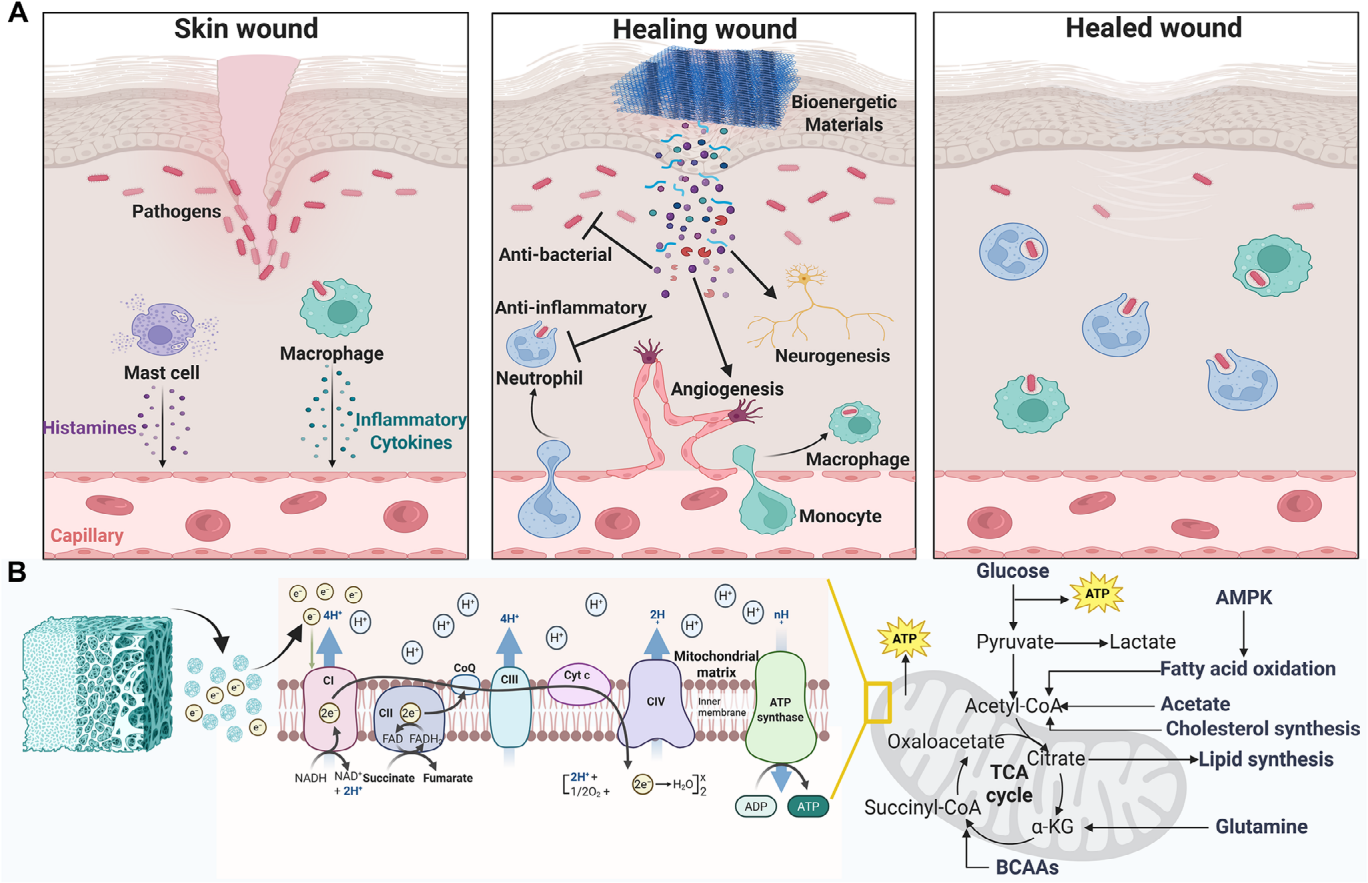
Bioenergetic materials promote wound healing through metabolic and mitochondrial regulation. (A) Schematic illustration of the wound healing process. In a skin wound, pathogens stimulate mast cells and macrophages to release histamines and inflammatory cytokines, driving inflammation. Application of bioenergetic materials enhances repair by exerting antibacterial and anti‐inflammatory effects, promoting angiogenesis, macrophage modulation, and neurogenesis, leading to accelerated closure and regeneration of the injured tissue. (B) Bioenergetic mechanisms underlying tissue repair. Bioenergetic materials enhance mitochondrial oxidative phosphorylation and increase ATP production by delivering key metabolites (e.g., TCA cycle intermediates), functional mitochondria, or redox‐active cofactors that facilitate electron flux through the electron transport chain. Concurrently, these interventions modulate central metabolic pathways, including glycolysis, TCA cycle, fatty acid oxidation, cholesterol and lipid synthesis, and amino acid metabolism, to rewire cellular energetics (Created with BioRender.com).

Bioenergetic materials are effective in treating chronic wounds, including diabetic foot ulcers and severe burns [154]. As discussed in previous sections, these advanced platforms promote tissue repair through the direct supplementation of ATP and TCA cycle intermediates [46, 63], the dynamic adjustment of local pH levels to modulate metabolic enzyme activity [155], the amelioration of hypoxic microenvironments [70, 78], and the remote regulation of metabolic processes through photothermal and piezoelectric effects [86, 87]. In this section, we systematically review these strategies to provide a comprehensive understanding of their distinct roles (Figure 4A), dynamic behaviors, and complex cellular interactions within the wound bed (Figure 4B).

Preventing infection and inhibiting bacterial growth are crucial for promoting wound healing [156]. The strategic design of bioenergetic materials that suppress bacterial metabolism represents a promising approach to combat antimicrobial resistance. These materials function by disrupting key energy‐producing pathways within bacteria, including cellular respiration and ATP synthesis [157]. By disrupting bacterial energy metabolism and limiting ATP availability, these materials interfere with essential cellular processes, resulting in bacteriostatic or bactericidal effects while reducing the likelihood of resistance development [157]. For example, Shi and colleagues engineered a nanostructured composite named TiOx@C from a carbon framework integrated with titanium oxide (TiOx) nanodots [158]. This nanocomposite exhibited antibacterial activity through both physical disruption and interference with bacterial electron transport chains. Specifically, the fibrous carbon component of TiOx@C enabled penetration of the bacterial envelope of *P. aeruginosa*, which possesses a thinner peptidoglycan layer compared with *S. aureus*. This penetration resulted in structural collapse, oxidative damage, and leakage of cellular contents [158]. For thick‐walled *S. aureus*, the composite disrupted metabolic activity by interfering with the electron transport chain, ultimately depleting energy supply and causing cell death. In vivo tests demonstrated a 97% reduction in bacterial load within infected wounds and accelerated healing in a murine model, highlighting its potential as a non‐antibiotic therapy [158]. Similarly, mesoporous catechin nanoparticles developed by Zhao and co‐workers exert antibacterial effects via membrane disruption and metabolic interference in bacteria [159]. These nanoparticles have well‐defined spherical morphology and high surface area, demonstrating exceptional inherent antibacterial properties through a synergistic mechanism whereby the mesoporous structure physically disrupted bacterial membranes while the catechin molecules chemically interfered with metabolic pathways, achieving the complete inhibition of *S. aureus* at low concentrations in both in vitro and in vivo models [159].

Regulating energy metabolism to promote the proliferation and migration of epithelial cells represents another effective strategy for enhancing wound healing [160]. Demonstrating this, Liu and colleagues found that two‐dimensional black phosphorus nanosheets (BPNSs) activated the JAK‐STAT‐OAS signaling pathway, thereby improving cellular function and mitochondrial energy metabolism in endothelial cells [161]. Subsequent in vivo studies showed that BPNS treatment promoted wound repair in a rat model of large full‐thickness wound through multifaceted biological effects, including enhanced anti‐inflammatory responses, angiogenesis, collagen deposition, and re‐epithelialization [161].

Compared with those for bone or cartilage repair, bioenergetic materials targeting skin wound healing are currently focused on infection control, redox normalization, and restoration of a metabolically permissive wound bed. This emphasis is particularly relevant for diabetic ulcers, which are characterized by hyperglycemia, persistent inflammation, mitochondrial dysfunction, excess ROS, impaired angiogenesis, and bacterial burden. Accordingly, recent studies have shown that [158] TiO_x_@C nanocomposites, and black‐phosphorus‐based systems [161] can improve wound repair by augmenting cellular functions, reducing bacterial metabolic fitness, rebalancing macrophage phenotypes, and restoring mitochondrial or endothelial functions. The strategies discussed in this subsection show strong clinical relevance, as chronic wounds are metabolically dysregulated lesions for which local bioenergetic rescue is conceptually well aligned with the underlying pathology. However, the evidence base remains predominantly limited to rodent or short‐term porcine models, and many studies prioritize closure speed over recurrence, scar quality, tensile strength, and durable re‐epithelial barrier restoration. To facilitate successful clinical translation, future research should place greater emphasis on long‐term diabetic models, polymicrobial biofilm challenges, and off‐the‐shelf dressing formulations.

### 3.4 Neural Regeneration

**FIGURE 5:**
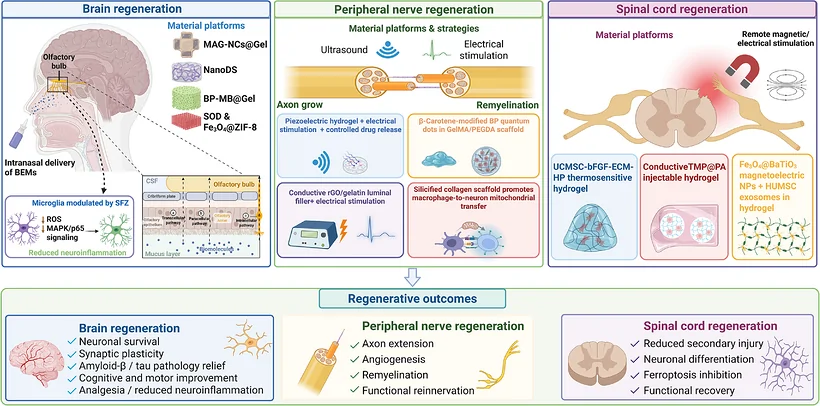
Bioenergetic materials for neural regeneration. For brain regeneration, representative BEM platforms include intranasal delivery systems designed to partially bypass the blood‐brain barrier (BBB), such as magnolol nanocrystal‐loaded thermosensitive hydrogels (MAG‐NCs@Gel), carrier‐free NanoDS nanomodulators, black phosphorus‐methylene blue thermosensitive hydrogels (BP‐MB@Gel), and superoxide dismutase/Fe_3_O_4_‐loaded ZIF‐8 nanoenzymes (SFZ). These systems have been shown to improve mitochondrial function, restore ATP production, reduce oxidative stress, modulate microglial activation, and alleviate neuroinflammation. In peripheral nerve regeneration, various BEM‐based platforms have been utilized–‐including ultrasound‐responsive piezoelectric nanofiber hydrogel conduits, antioxidant black phosphorus quantum dot‐containing GelMA/PEGDA scaffolds, conductive reduced graphene oxide (rGO)/gelatin luminal fillers combined with electrical stimulation, and silicified collagen scaffolds that enhance macrophage‐to‐neuron mitochondrial transfer–‐to promote axonal extension, angiogenesis, remyelination, and functional reinnervation. For spinal cord injury (SCI) repair, representative BEM platforms include UCMSC‐bFGF‐ECM‐HP thermosensitive hydrogels, conductive TMP@PA injectable hydrogels, and Fe_3_O_4_@BaTiO_3_ magnetoelectric nanoparticle/exosome‐loaded hydrogels. These scaffolds have been found to restore mitochondrial homeostasis, suppress oxidative stress and ferroptosis, reconstruct electrophysiological continuity, and support neuronal differentiation and functional recovery. Collectively, BEMs coordinate energy metabolism, redox balance, immune responses, and electrophysiological repair to promote neural regeneration. (Created with BioRender.com).

Neural regeneration has long presented a formidable challenge in regenerative medicine [162]. The unique metabolic and neuro‐electrophysiological properties of neurons require highly specialized regulatory strategies [163]. In the present review, we primarily focus on brain regeneration within the CNS, while also considering peripheral nerve and spinal cord repair. For CNS, the presence of the blood‐brain barrier (BBB) greatly complicates drug delivery, limiting the efficiency of many therapeutic approaches [164]. In recent years, intranasal drug administration through the olfactory epithelium (OE) has emerged as a direct and highly efficient delivery route to the central nervous system, partially bypassing the BBB [165] (Figure 5). Furthermore, modulating intrinsic metabolic pathways is now recognized as a strategy to optimize mitochondrial function, energy availability, and redox balance within damaged neural tissues [166]. As such, targeted metabolic modulation has become an important approach to enhance neuronal survival and promote functional regeneration [167]. In this section, we discuss how such bioenergetic interventions operate, highlighting specific biomaterial platforms and their mechanisms.

Neurons have high energy demands to maintain ionic gradients and synaptic activity, with these requirements increasing even further during axonal regeneration [168]. During aging and in pathological states, the cellular milieu of the brain exhibits hallmark signs of compromised bioenergetics [169]. Providing metabolic fuels and improving mitochondrial function are therefore key regenerative strategies to support neuronal bioenergetics and functional recovery [170]. To improve mitochondrial function, Chen and colleagues developed a thermosensitive hydrogel composed of magnolol nanocrystals (MAG‐NCs) embedded in PNIPAM (MAG‐NCs@Gel) [171]. This gel exhibited higher viscoelasticity within the nasal cavity, facilitating the sustained delivery of therapeutics across the BBB. Once in the brain, MAG‐NCs accumulated in dopaminergic neurons, where they reduced ROS, restored ATP levels, and reversed mitochondrial dysfunction. In an MPTP‐induced Parkinson's disease (PD) murine model, this approach significantly alleviated motor deficits while maintaining excellent tissue safety [171]. To further enhance delivery efficiency, Chen and colleagues constructed a multifunctional intranasal delivery scaffold (BP‐MB@Gel) [172]. This system integrated black phosphorus nanosheets with methylene blue, embedding them within a thermosensitive hydrogel formed from carboxymethyl chitosan and aldehyde‐modified Pluronic F127. Following intranasal administration, the gradual release of BP‐MB successfully restored the mitochondrial function of resident neurons and suppressed neuroinflammation. In AD mouse models, this synergistic bioenergetic strategy led to significant cognitive improvements, offering a promising platform for brain‐targeted therapy [172]. Beyond small‐molecule delivery, the direct transfer of mitochondria from macrophages to neurons can provide stressed neural cells with additional bioenergetic support, thereby protecting peripheral nerves and promoting neural regrowth [125]. As discussed previously, Niu and colleagues demonstrated that bioactive silicon can restore macrophage mitochondrial function and facilitate the active transfer of healthy mitochondria to neuronal cells via microvesicles [125]. By incorporating a Drp1‐Fis1 interaction inhibitor into a silicified collagen scaffold, they further strengthened this intercellular mitochondrial trafficking, ultimately leading to improved axonal extension and functional reinnervation within the defect site [125].

Rationally designed BEMs have also been successfully applied to peripheral nerve repair. Xu and colleagues developed aligned piezoelectric nanofiber‐derived hydrogel conduits for ultrasound‐triggered electrical stimulation and controlled drug release [173]. These nerve guidance conduits promoted axonal regeneration and functional recovery in rats with long sciatic nerve defects. Shen and colleagues embedded antioxidant β‐carotene‐modified black phosphorus quantum dots into a GelMA/PEGDA scaffold and demonstrated improved angiogenesis, inflammatory regulation, axon remyelination, and functional recovery in both rat and beagle peripheral nerve injury models [174]. Park and coworkers further showed that an injectable conductive rGO/gelatin luminal filler, combined with electrical stimulation, could drive nerve regrowth and myelination at levels comparable to autograft‐induced repair in a rat defect model [175]. Collectively, these findings indicate that BEMs designed for peripheral nerve repair can integrate metabolic signaling, oxidative‐stress control, and electroactive guidance within a single platform. Nevertheless, most existing strategies have only been validated in small animals, and several challenges remain, including the blood‐nerve/perineurial barriers, long‐gap repair durability, device dependence, and scalable manufacturing of multifunctional conduits.

In the context of spinal cord injury repair, BEMs have been predominantly used to rescue secondary injury‐associated metabolic disruptions, rather than to directly replace damaged or lost tissues. Li and coworkers designed a UCMSC‐bFGF‐ECM‐HP thermosensitive hydrogel, comprising umbilical cord MSCs and basic fibroblast growth factor immobilized in a bioactive polymer of heparin‐poloxamer and spinal cord extracellular matrix. This system was shown to improve mitochondrial function, promote mitochondrial fusion, reduce pathological fragmentation, and lower MDA, LDH, and ROS, thereby supporting spinal cord repair in mice [176]. A recent conductive TMP@PA injectable hydrogel combined electrical conduction pathway reconstruction with sustained tetramethylpyrazine release to inhibit YAP‐regulated, NCOA4‐mediated ferritinophagy and ferroptosis [177]. Liu and collaborators combined Fe_3_O_4_@BaTiO_3_ magnetoelectric nanoparticles with human umbilical MSC‐derived exosomes in a hydrogel, enabling remote noninvasive electrical stimulation to simultaneously enhance neuronal differentiation and immunoregulation after SCI [178]. The shared advantage of these SCI platforms lies in their ability to concurrently address multiple metabolic failure axes, including oxidative stress, mitochondrial dysfunction, ferroptosis, and loss of electrophysiological continuity. However, several limitations persist: most evidence comes from acute rodent SCI models rather than chronic scar‐dominated lesions, the long‐term persistence and safety of conductive or magnetoelectric components remain unclear, and manufacturing complexities for gene‐ and exosome‐based therapies pose substantial translational barriers.

Across central and peripheral nerve repair, neural BEMs are now converging on a shared design rationale: repair is enhanced when biomaterials restore mitochondrial fitness, counter oxidative stress, provide electroactive cues, or reprogram immune‐neural crosstalk, rather than relying on single neurotrophic cues. This subsection has highlighted the growing diversity of these complementary strategies. Nonetheless, the evidence base remains fragmented and predominantly preclinical, and clinical translation faces distinct hurdles. Brain‐directed therapies are hampered by BBB/BSCB barriers and dosing variability. Platforms for peripheral nerve repair have yet to demonstrate efficacy in long‐gap injuries and overcome the blood‐nerve barrier. Most SCI treatment data were derived from acute rodent models. Added to these are unresolved questions regarding material persistence, manufacturing scale‑up for exosome/genetic cargoes, and the absence of standardized metabolic outcome measures. Ultimately, neural BEMs hold considerable scientific appeal, but meaningful translational progress will require larger animal models, longer follow‐up, and a sharper focus on realistic manufacturability.

The exemplary progress in BEM‐fueled tissue regeneration discussed in this section span bone, cartilage and joint, skin, and neural repair applications, and is well aligned with the core regulatory mechanisms outlined in Section 2. By enabling metabolic reprogramming, modern BEMs have evolved beyond passive scaffolds into active metabolic modulators. Their demonstrated benefits include enhanced mitochondrial function, augmented local ATP bioavailability, and coordinated regulation of complex metabolic signaling pathways, collectively shifting compromised cells toward a highly anabolic and regenerative state. Together, these studies underscore the clinical promise of bioenergetic interventions, providing a comprehensive perspective on how tailored material designs and molecular targeting strategies can be integrated to achieve robust regenerative outcomes across diverse biological systems.

## 4 Conclusions and Future Perspectives

BEMs represent a fundamentally distinct class of regenerative biomaterials that directly target the bioenergetic processes governing cellular function, rather than serving solely as structural scaffolds or delivery vehicles for biological factors [22]. A central conclusion emerging from the current literature is that effective tissue regeneration requires more than transient enhancement of ATP production. Instead, successful regeneration depends on coordinated bioenergetic regulation that integrates mitochondrial function, redox homeostasis, inflammation resolution, angiogenesis, extracellular matrix remodeling, cell differentiation, and tissue‐specific functional maturation within the evolving injury microenvironment [20, 179]. Collectively, the studies reviewed here demonstrate that BEMs achieve their therapeutic effects by reshaping the local bioenergetic microenvironment through multiple complementary mechanisms, including metabolic substrate supplementation, enzyme‐mediated metabolic regulation, oxygen and redox modulation, mitochondrial restoration, and biochemical stimulation. Accordingly, BEMs should be regarded not simply as energy‐supplying materials, but as active regulators of cellular bioenergetics that establish the metabolic conditions necessary for endogenous tissue regeneration.

**FIGURE 6:**
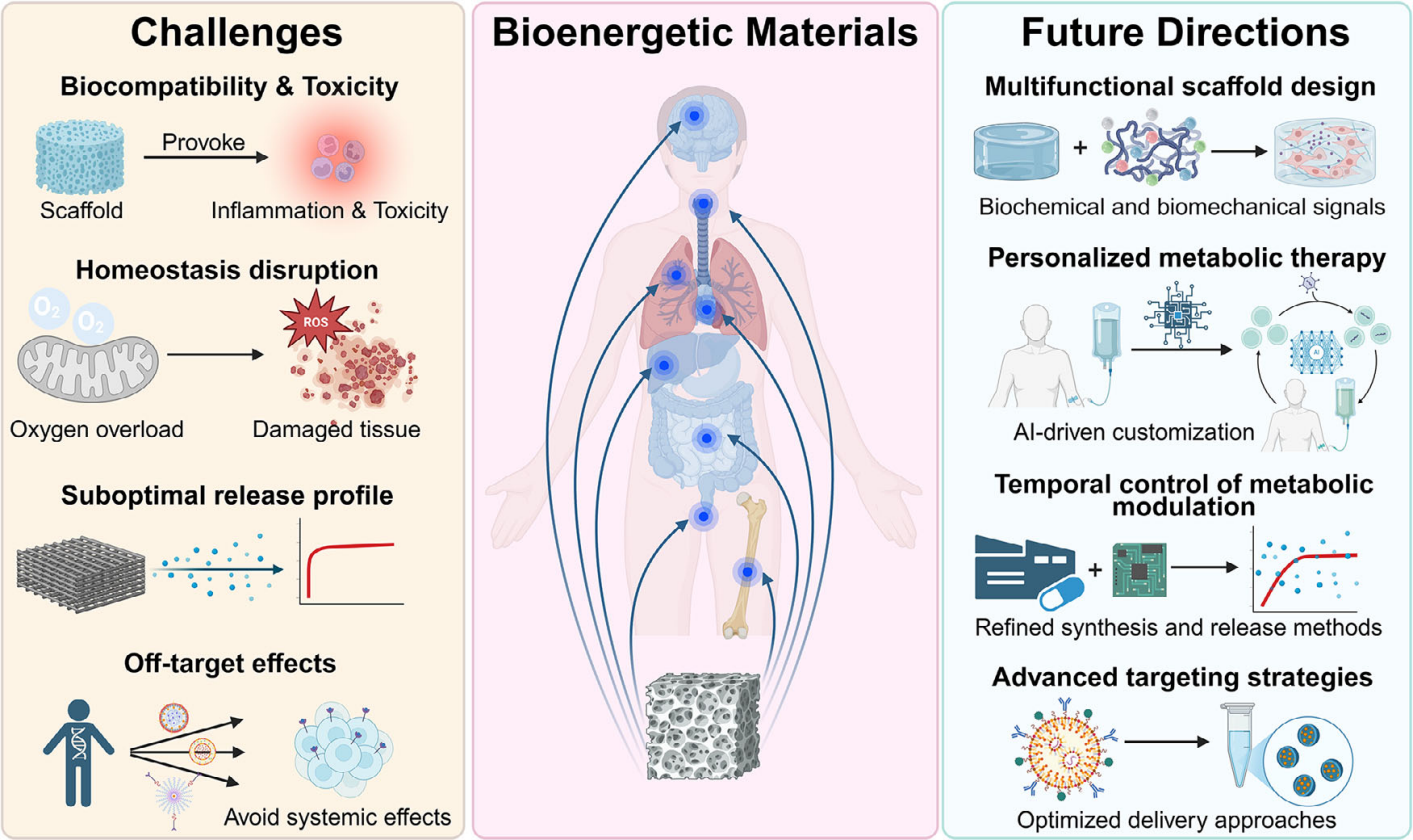
Challenges and future directions of BEMs in regenerative medicine. Major challenges include insufficient biocompatibility and toxicity concerns, disruption of physiological homeostasis, suboptimal biomolecule release kinetics that limits effective metabolic stimulation, and off‐target effects. BEMs, with bioactive, metabolism‐regulating properties, integrate structural support with metabolic cues to modulate cellular bioenergetics across multiple tissues and organs. Future directions for multifunctional scaffold development include integration of biochemical and biomechanical signals, personalized metabolic therapies enabled by patient‐specific metabolic profiling and AI‐driven customization, refined temporal control of metabolic modulation through advanced synthesis and release strategies, and advanced targeting approaches to improve delivery precision while minimizing systemic side effects (Created with BioRender.com).

Beyond regenerative applications discussed in this review, bioenergetic materials are rapidly gaining attention in other fields, particularly oncology, where modulation of cellular bioenergetics and the tumor microenvironment has emerged as a promising therapeutic strategy [180]. Nevertheless, the clinical transition of BEMs remains in its infancy. Unlike conventional biomaterials, BEMs must achieve precise spatiotemporal regulation of cellular bioenergetics while avoiding metabolic overstimulation, insufficient bioenergetic support, off‐target metabolic effects, systemic toxicity, and inappropriate activation of non‐target cell populations. These challenges are further compounded by the dynamic metabolic landscape of tissue repair, where cellular energy and metabolic phenotypes evolve throughout the regenerative process. Therefore, next‐generation BEMs should move beyond generalized metabolic enhancement toward tissue‐specific, stage‐dependent, and cell‐selective bioenergetic modulation capable of adapting to the changing regenerative microenvironment (Figure 6).

### 4.1 Biocompatibility and Long‐Term Biosafety

Because bioenergetic materials are designed to actively modulate cellular metabolism, their long‐term safety extends beyond the conventional biocompatibility requirements of regenerative biomaterials. In addition to the biological effects of degradation products, released metabolites, ions, redox‐active components, and bioelectrical or photothermal outputs, BEMs must preserve metabolic homeostasis without inducing unintended local or systemic metabolic disturbances. Accordingly, biosafety evaluation should extend beyond standard cytotoxicity and histocompatibility assays to include chronic inflammation, fibrosis, oxidative stress, mitochondrial integrity, immunometabolic responses, and systemic metabolic alterations following prolonged implantation or repeated exposure.

Biodegradable polymers illustrate why metabolic compatibility should become an integral component of BEM safety assessment. Polylactide (PLA), one of the most widely used biodegradable polymers in tissue engineering, degrades into D‐ and L‐lactic acid, which have been associated with adverse immune responses and fibrosis in both preclinical models and human studies [181, 182]. Mechanistic investigations further demonstrated that these degradation products reprogram macrophage and fibroblast metabolism by enhancing glycolytic activity, thereby promoting pro‐inflammatory cytokine production and fibrotic remodeling [183]. These findings raise concerns regarding the long‐term viability of PLA‐based implants, particularly in load‐bearing or soft tissue applications where sustained degradation may contribute to chronic inflammation, impaired tissue integration, and implant failure. To mitigate these adverse effects, Contag and colleagues investigated the addition of small‐molecule inhibitors targeting distinct steps in the glycolytic pathway to modulate the metabolic state of macrophages and fibroblasts exposed to PLA [183]. These inhibitors, including 3‐(3‐pyridinyl)‐1‐(4‐pyridinyl)‐2‐propen‐1‐one, 2‐deoxyglucose, and aminooxyacetic acid, successfully stimulated anti‐inflammatory cytokine expression by preventing the PLA‐induced metabolic reprogramming and altered cellular bioenergetics in a dose‐dependent manner [183]. Moving forward, combining such pharmacological interventions with advanced surface modifications and the precise dose optimization of bioenergetic payloads will be critical. Carefully titrating the amount of metabolic stimulant to avoid overshooting physiological thresholds is essential for preventing unwanted cellular responses.

Metabolite‐based BEMs offer an inherent advantage because many of their bioactive components, including citrate, succinate, α‐KG, and malate, are endogenous metabolic intermediates that can be readily incorporated into physiological metabolic pathways. However, endogenous origin does not necessarily equate to biological safety. Supraphysiological concentrations or prolonged exposure to these metabolites may perturb metabolic homeostasis and elicit context‐dependent effects on immune activation, epigenetic regulation, osteoclast function, and inflammatory signaling. Future studies should establish the therapeutic window for each bioenergetic cue by systematically defining the optimal local concentration, spatiotemporal release profile, metabolic clearance, and cell type‐specific responses required to maximize regenerative efficacy while minimizing off‐target effects.

### 4.2 Controlled Release and Spatiotemporal Regulation

The therapeutic efficacy of BEMs depends not only on the composition of the bioenergetic payload but also on its spatiotemporal delivery. Tissue repair is a dynamic process encompassing overlapping inflammatory, proliferative, angiogenic, matrix‐remodeling, and maturation phases, each characterized by distinct metabolic requirements. Consequently, bioenergetic cues should be delivered in a manner that mirrors these evolving demands. A burst release of oxygen, bioactive ions, or metabolites may provide transient metabolic stimulation but fail to sustain long‐term regeneration, whereas prolonged or excessive release may disrupt metabolic homeostasis and impair tissue repair. For example, uncontrolled oxygen generation from peroxide‐based materials can rapidly exhaust oxygen reserves and produce excessive ROS, resulting in oxidative damage to surrounding tissues [20]. Incorporation of antioxidant enzymes such as catalase into oxygen‐releasing systems has therefore emerged as an effective strategy to regulate oxygen delivery while limiting ROS‐mediated toxicity [20]. Additionally, material design strategies that regulate polymer composition, crosslinking density, scaffold architecture, and degradation kinetics have emerged as effective approaches for achieving sustained bioenergetic delivery [184]. For example, sustained delivery of endogenous metabolic intermediates from the succinate‐releasing scaffold discussed previously provides prolonged bioenergetic support while maintaining compatibility with physiological metabolic pathways [24].

Looking forward, future BEMs should therefore incorporate stage‐specific and adaptive release profiles that match the evolving metabolic demands of tissue repair. During the early inflammatory phase, bioenergetic interventions may preferentially reduce oxidative stress, improve oxygen availability, and promote resolution of inflammation, whereas subsequent stages may require sustained metabolic support for extracellular matrix deposition, angiogenic and vascular maturation, mineralization, axonal extension, and functional tissue remodeling [72]. Stimuli‐responsive biomaterials capable of responding to changes in pH, ROS, enzymatic activity, hypoxia, or other pathological cues represent a strategy for achieving this spatiotemporal precision. However, the performance of these systems must be validated in physiologically relevant models, as the metabolic and biochemical complexity of human injury microenvironments is unlikely to be fully recapitulated in simplified in vitro or small‐animal models. The development of New Alternative Methodologies, including organs‐on‐chips (OoCs), provides new opportunities for further research in this field [185, 186, 187].

### 4.3 Targeted Delivery and Tissue‐Specific Precision

Effective bioenergetic modulation requires precise delivery to the appropriate tissues, cell populations, and intracellular compartments while minimizing unintended systemic effects [188]. Unlike traditional growth factors or morphogens, many bioenergetic agents are small, diffusible metabolites that can readily distribute beyond the target site, raising the possibility of off‐target metabolic reprogramming and unintended activation of neighboring or distant cell populations [189]. For instance, a systemic infusion of succinate could inadvertently fuel the proliferation of unwanted cell types or inappropriately activate off‐target immune cells via widespread succinate receptor binding [190]. Therefore, precise spatial control is essential to ensure that the bioenergetic material acts primarily at the site of injury or defect. One straightforward approach is the local implantation or injection of the bioenergetic construct. By placing the material directly into the defect site, the bioenergetic boost is physically confined to the target microenvironment. This principle is illustrated by the previously discussed α‐KG‐loaded hydrogel, which was implanted directly into diabetic bone defects to localize metabolic and immunomodulatory effects while minimizing systemic exposure [39, 85]. Future BEMs should further integrate tissue‐targeting ligands, cell‐selective delivery strategies, and organelle‐specific targeting technologies to achieve precise bioenergetic regulation with maximal therapeutic efficacy and minimal off‐target effects.

Beyond anatomical localization, molecular targeting strategies provide an additional level of precision by directing bioenergetic materials toward specific cell populations and intracellular compartments. Functionalization of biomaterial carriers with tissue‐ or cell‐specific ligands facilitates the precise homing of bioenergetic payloads to particular cell populations. As discussed previously, coating NTUs with chondrocyte membranes enabled biomimetic recognition and preferential fusion with host chondrocytes, thereby facilitating localized delivery of metabolic cargo to degenerative cartilage [77]. Future BEMs may further exploit biomimetic membrane coatings, antibody‐conjugated carriers, or receptor‐targeting ligands to improve tissue and cell specificity. Cell‐mediated delivery also represents an attractive strategy, whereby endogenous or engineered stem cells and immune cells serve as living carriers that actively home to sites of injury and deliver bioenergetic payloads in situ [191, 192]. At the subcellular level, therapeutic efficacy further depends on precise organelle targeting, particularly for metabolic modulators that act within mitochondria. Incorporating mitochondria‐targeting peptides or related intracellular delivery technologies into biomaterial platforms may substantially improve bioenergetic efficiency by directing therapeutic cargo to its primary site of action [74]. Collectively, integrating tissue‐, cell‐, and organelle‐specific targeting strategies will be critical for maximizing the therapeutic precision of BEMs while minimizing systemic metabolic perturbations.

### 4.4 Integration With Biochemical, Biomechanical, and Bioelectrical Cues

Cellular bioenergetics operates within a highly integrated regulatory network and should not be considered independently of other regenerative cues. Cellular responses to bioenergetic modulation are simultaneously shaped by biochemical signaling, extracellular matrix composition and mechanics, immune‐cell interactions, oxygen availability, mechanical loading, and endogenous bioelectrical activity. Consequently, the therapeutic efficacy of BEMs will depend not only on their ability to restore cellular energy metabolism but also on their capacity to coordinate with these complementary microenvironment signals. Integrating bioenergetic modulation with biochemical, biomechanical, and bioelectrical signaling represents another important direction for the development of next‐generation BEMs.

Electroactive materials, including conductive, piezoelectric, magnetoelectric, and triboelectric systems, illustrate this concept by converting endogenous or externally applied physical stimuli into bioelectrical signals that regulate cellular behavior. For example, conductive nerve guidance conduits combined with wireless electrical or magnetoelectric stimulation promoted axonal regeneration, myelination, and functional recovery in preclinical models [193]. Likewise, multifunctional hydrogel platforms could simultaneously deliver bioenergetic metabolites, provide structural support for tissue remodeling, and present biochemical or mechanical cues that direct cell differentiation [194]. Such integrated strategies more closely recapitulate the native regenerative microenvironment and are likely to produce more robust and functionally mature tissue repair than modulation of any single pathway alone.

### 4.5 Personalized and Disease Stage‐Specific Bioenergetic Therapy

A major challenge for future BEM development is biological heterogeneity. The metabolic state of injured tissues varies across disease types, patient age, inflammatory status, oxygen availability, vascular supply, infection burden, and repair stage. A metabolic intervention that benefits one tissue or disease stage may be ineffective or even harmful in another context. Therefore, patients will inevitably require tailored bioenergetic interventions. The tissues of aged patients often exhibit distinct metabolic vulnerabilities, potentially requiring a more substantial or prolonged bioenergetic boost compared to younger cohorts [195]. To address this heterogeneity, comprehensive metabolic profiling of injured tissues could be utilized to guide the precise selection and dosing of bioenergetic materials [196]. For example, aged bone MSCs often show impaired mitochondrial function and reduced osteogenic potential, which may require strategies aimed at mitochondrial rejuvenation or metabolic reactivation. Diabetic wounds are characterized by persistent inflammation, hypoxia, oxidative stress, impaired angiogenesis, and infection susceptibility, suggesting the need for combined oxygenation, redox control, antibacterial activity, and metabolic support. Osteoarthritic cartilage involves mitochondrial dysfunction, oxidative stress, abnormal glycolysis, and matrix catabolism, indicating that chondrocyte‐centered therapy alone may not be sufficient. Neural injury requires not only neuronal survival but also axonal extension, remyelination, glial regulation, synaptic reconnection, and restoration of electrophysiological function.

To accurately evaluate BEM efficacy, new metabolic biomarkers must be developed, coupled with advanced assessment methods, including imaging modalities such as NADH/FAD autofluorescence, PET, and hyperpolarized MRI. Complementing these approaches, multi‐omics and functional profiling spanning metabolomics, transcriptomics, single‐cell analysis, spatial profiling, mitochondrial function assays, and non‐invasive metabolic imaging could all help identify tissue‐specific metabolic vulnerabilities. By integrating advanced diagnostic technologies, researchers could design customized scaffolds capable of precisely delivering the specific substrates or cofactors deficient in an individual patient's cells. Such personalized approaches stand to maximize the therapeutic benefits of metabolic modulation while curbing off‐target systemic effects. Moreover, as the role of metabolism in fibrosis and inflammation becomes better understood, the delivery of these modulators could be temporally calibrated, such that specific bioenergetic cues can be deployed at defined phases of the healing process to actively promote tissue regeneration and suppress pathological scarring.

### 4.6 Regulatory Science and Clinical Translation Pathways

Despite rapid preclinical progress, the translation of BEMs into clinically viable regenerative therapies remains nascent. A major obstacle is their regulatory heterogeneity. Depending on their composition and primary mode of action, they may fall under the purview of drugs, biologics, medical devices, tissue‐engineered products, advanced therapy medicinal products, or combination products. This ambiguity becomes particularly critical for complex systems. Regulatory classification should therefore be considered at the outset of product development, rather than deferred until efficacy has been demonstrated, because classification determines the subsequent requirements for manufacturing control, preclinical safety testing, clinical trial design, and regulatory approval.

A second challenge lies in establishing robust chemistry, manufacturing, and control strategies. For conventional biomaterials, physical and chemical characterization often suffice to ensure product consistency. For BEMs, however, therapeutic activity hinges on dynamic, function‐driven properties, including oxygen generation, ATP production, redox buffering, metabolic substrate release, mitochondrial respiration, enzymatic activity, and stimulus‐responsive electrical output. Batch release criteria must therefore extend beyond conventional metrics such as particle size, morphology, composition, or mechanical properties. Going forward, the field should define critical quality attributes that directly reflect the intended mechanism of action and develop quantitative potency assays that robustly correlate these attributes with biological efficacy.

A further challenge in the preclinical‐to‐clinical transition is the need for more predictive models and clinically meaningful endpoints. Most studies have evaluated BEMs in small, acute, and relatively homogeneous defect models, yet clinical regeneration typically unfolds in aged patients burdened by chronic inflammation, vascular dysfunction, metabolic disease, or extensive tissue loss. These comorbidities can profoundly alter cellular bioenergetics and, consequently, modify tissue response to metabolic intervention. Future preclinical programs should therefore incorporate disease‐relevant large animal models, clinically translatable administration protocols, dose‐ranging studies, biodistribution and degradation analyses, systemic metabolic safety assessment, immunotoxicity evaluation, and long‐term evaluation of tissue structure and function.

A practical clinical translation pathway for BEMs should therefore begin with early regulatory classification and identification of the primary mode of action, followed by quality‐by‐design manufacturing development, definition of critical quality attributes, and establishment of mechanism‐related potency and release assays. Standardized pharmacology, biodistribution, degradation, and long‐term safety studies should then precede first‐in‐human trials. Early‐phase clinical studies should prioritize safety, feasibility, dose or stimulation window optimization, and biomarker‐based confirmation of target engagement. Subsequent efficacy trials should employ indication‐specific functional endpoints and incorporate appropriate patient stratification, particularly when key variables, such as age, diabetes, ischemia, or chronic inflammation, may influence baseline bioenergetic states and modulate treatment responsiveness. For eligible regenerative medicine products, expedited regulatory pathways can facilitate interaction with regulators and streamline development planning; however, such mechanisms do not obviate the need for rigorous product characterization, manufacturing consistency, and robust clinical evidence.

### 4.7 Limitations and Future Perspectives

This review has several limitations. First, as a narrative review rather than a systematic review or meta‐analysis, it draws on representative studies to outline the development of BEMs in tissue regeneration. Due to space constraints, it could not exhaustively cover all relevant published literature. Second, the studies surveyed are highly heterogeneous, varying widely in material composition, active bioenergetic components, release kinetics, target tissue, disease model, administration route, observation period, and evaluation endpoints. Moreover, while many studies assessed tissue repair through histology, imaging, and functional recovery, detailed metabolic assays were not consistently performed. This heterogeneity and the frequent absence of direct metabolic readouts complicate direct cross‐study comparisons and make it difficult to ascertain whether the observed regenerative effects stem primarily from bioenergetic modulation or from ancillary biological effects of the material itself. Consequently, the conclusions presented here should be interpreted as a conceptual and mechanistic synthesis rather than a quantitative benchmark of therapeutic efficacy. These limitations also highlight the need for a more consistent evidentiary standard for future BEM studies.

To improve mechanistic rigor and comparability across future studies, we propose a minimum evidence framework for attributing regenerative effects to bioenergetic modulation. First, the material should be quantitatively characterized for the bioenergetic function relevant to its proposed mechanism, such as metabolite or oxygen release, enzymatic activity, ATP/NADPH generation, mitochondrial transfer, or electrical output. Second, at least one direct cellular bioenergetic endpoint should be measured in the relevant recipient cells or tissue, including ATP production or content, mitochondrial membrane potential, mitochondrial respiration/oxidative phosphorylation, glycolytic energetic flux, or another mechanism‐appropriate measure of energy‐generating metabolic flux. Measurements of ROS, antioxidant activity, metabolic gene expression, mitochondrial morphology, inflammatory signaling, or tissue repair alone should not be considered sufficient evidence of bioenergetic modulation. Third, the measured energetic change should be associated with a tissue‐relevant regenerative outcome, such as cell proliferation or differentiation, extracellular matrix synthesis, angiogenesis, axonal growth, or functional tissue recovery. Ideally, causal evidence should further be established by perturbing the proposed bioenergetic pathway or by demonstrating that blockade of the energetic response attenuates the regenerative benefit. Together, this material function–bioenergetic response–regenerative outcome framework would provide a more consistent evidentiary basis for distinguishing bona fide bioenergetic mechanisms from secondary metabolic associations and facilitate meaningful comparison across BEM platforms.

Overall, this review positions cellular metabolism as an active and engineerable component of tissue repair, rather than a passive byproduct of regeneration. BEMs offer a compelling framework for engineering regenerative microenvironments through targeted metabolic regulation. Their future success, however, will hinge on a paradigm shift from broad metabolic stimulation toward precise spatiotemporal control over when, where, and how bioenergetic cues are delivered. By integrating metabolic modulation with tissue‐specific targeting, dynamic release kinetics, mechanical adaptation, bioelectrical regulation, and rigorous clinical validation, BEMs hold the potential to emerge as a transformative class of next‐generation biomaterials for repairing metabolically compromised tissues.

## Author Contributions

**Yuchen He**: conceptualization, writing – original draft, investigation, formal analysis, methodology, data curation, funding acquisition. **Yuwen Wang**: formal analysis, writing – review and editing, visualization. **Jonathan F. Gong**: formal analysis, writing – review and editing. **Yiting Lei**: formal analysis, writing – review and editing. **Fei Jin**: formal analysis, writing – review and editing. **Weihong Zhu**: funding acquisition, writing – review and editing, project administration, resources. **Rocky S. Tuan**: project administration, writing – review and editing, funding acquisition. **Zhong Alan Li**: conceptualization, writing – review and editing, formal analysis, project administration, resources, funding acquisition, software.

## Conflicts of Interest

The authors declare no conflicts of interest.

## Data Availability

The data that support the findings of this study are available from the corresponding author upon reasonable request.
